# Innate Lymphoid Cells in Tissue Homeostasis and Diseases

**DOI:** 10.1002/mco2.70743

**Published:** 2026-04-21

**Authors:** Zhenzhen Zhan, Heyang Sun, Chenning Li, Qianya Hong, Shuainan Zhu, Ying Yu, Hao Zhang, Kefang Guo

**Affiliations:** ^1^ Department of Anesthesiology Zhongshan Hospital Fudan University Shanghai China; ^2^ Shanghai Key Laboratory of Perioperative Stress and Protection Shanghai China; ^3^ Department of Anesthesiology Shanghai Minhang Hospital Shanghai China

**Keywords:** diseases, homeostasis, immunotherapy, innate lymphoid cells, sepsis

## Abstract

Innate lymphoid cells (ILCs) are tissue‐resident immune sentinels that play pivotal roles in maintaining tissue homeostasis, orchestrating immune responses, and modulating metabolic balance. They rapidly respond to environmental cues and interplay with other immune cells, thereby mediating host defense and facilitating tissue repair. However, dysregulation of ILC responses is increasingly implicated in the pathogenesis of a broad spectrum of diseases. This review provides a comprehensive overview of ILC biology, beginning with their classification, plasticity, and homeostatic functions. We then dissect the complex, dual roles of ILCs across various pathological conditions. Using sepsis as a paradigmatic example of immune dysregulation, we illustrate how ILCs orchestrate both protective immunity and pathological role in a context‐dependent manner. Furthermore, we extend the discussion to cancer, chronic inflammatory diseases, and metabolic disorders, highlighting the tissue‐specific functions of ILC subsets. Finally, we synthesize emerging ILC‐targeted therapeutic strategies and future research directions, proposing that a nuanced understanding of ILC biology is essential for developing novel immunotherapies aimed at restoring immune homeostasis in human diseases.

## Graphical abstract

Innate lymphoid cells (ILCs) are a heterogeneous group of immune cells with phenotypic and functional plasticity. ILCs dynamically regulate various immune cell types and play a crucial role in the pathophysiological processes of specific organs during sepsis. Targeting ILCs is a promising strategy for treating sepsis.

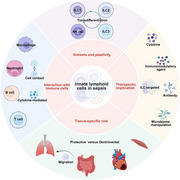

## 1 Introduction

Innate lymphoid cells (ILCs) have emerged as pivotal components of the innate immune system since their classification as distinct lineages over a decade ago [1]. Unlike T and B lymphocytes, ILCs do not express rearranged antigen receptors or rely on antigen presentation for activation; instead, they respond rapidly to alarmins, tissue‐derived signals, and environmental cues [1]. ILCs are strategically distributed throughout peripheral tissues and enriched in the mucosal barrier, where they orchestrate tissue homeostasis and serve as first responders to pathogen invasion, tissue damage, and metabolic alterations. They bridge innate and adaptive immunity through cytokine production and direct cellular interactions [2]. ILCs are broadly categorized into several subgroups—Group 1 ILCs, Group 2 ILCs, Group 3 ILCs, and regulatory ILCs (ILCregs)—each defined by distinct transcription factors (TFs) and effector cytokine profiles that functionally mirror CD4^+^ helper (Th) and CD8^+^ cytotoxic T lymphocytes [3]. Under homeostatic conditions, ILCs are indispensable for maintaining tissue integrity, facilitating organogenesis, promoting barrier immunity, and orchestrating metabolic balance. However, their dysregulation is increasingly implicated in the pathogenesis of a wide spectrum of diseases, highlighting their dual role as guardians of health and perpetrators of pathology [4].

Despite their recognized importance in homeostasis, the precise and often context‐dependent functions of ILCs across various human diseases remain only partially understood. This review is motivated by the need to elucidate the rapidly expanding knowledge of ILC biology, from their basic development and homeostatic functions to their complex roles in pathological states. We aim to move beyond a mere catalog of ILC activities to offer an integrated perspective on their immunoregulatory networks. A particular highlight of our discussion is the in‐depth analysis of sepsis as a paradigm disease. Sepsis represents a unique and compelling model for studying ILC dynamics because of its hallmark features: a dramatic cytokine storm, severe tissue damage, profound immune dysregulation encompassing both hyperinflammation and subsequent immunosuppression, and multiorgan dysfunction [5, 6]. The rapid and dynamic nature of sepsis mirrors the core characteristics of ILCs—their swift responsiveness, functional plasticity, and extensive cellular crosstalk [7]. Therefore, dissecting the intricate role of ILCs in sepsis not only provides insights into the pathophysiology of this heterogeneous syndrome but also serves as a powerful lens through which to understand the fundamental principles of ILC behavior in other acute and chronic inflammatory diseases.

This review is structured to first provide a foundational overview of the development, key characteristics, and plasticity of major ILC subsets. We then delve into their critical functions in maintaining tissue homeostasis, including immune defense, tissue repair, and metabolic regulation. The core of the article examines the multifaceted roles of ILCs in diseases, with sepsis serving as a central paradigm to illustrate their interactions with other immune cells and their tissue‐specific effects. We further extend the discussion to other pathological conditions, including cancer, chronic inflammatory diseases, and metabolic disorders, thereby situating the insights gained from the sepsis paradigm within a broader immunological context. Finally, we summarize emerging ILC‐targeted therapeutic strategies and conclude with future perspectives, aiming to bridge basic science with clinical translation and inspire novel immunotherapeutic interventions.

## 2 Overview of ILC Subsets

**FIGURE 1:**
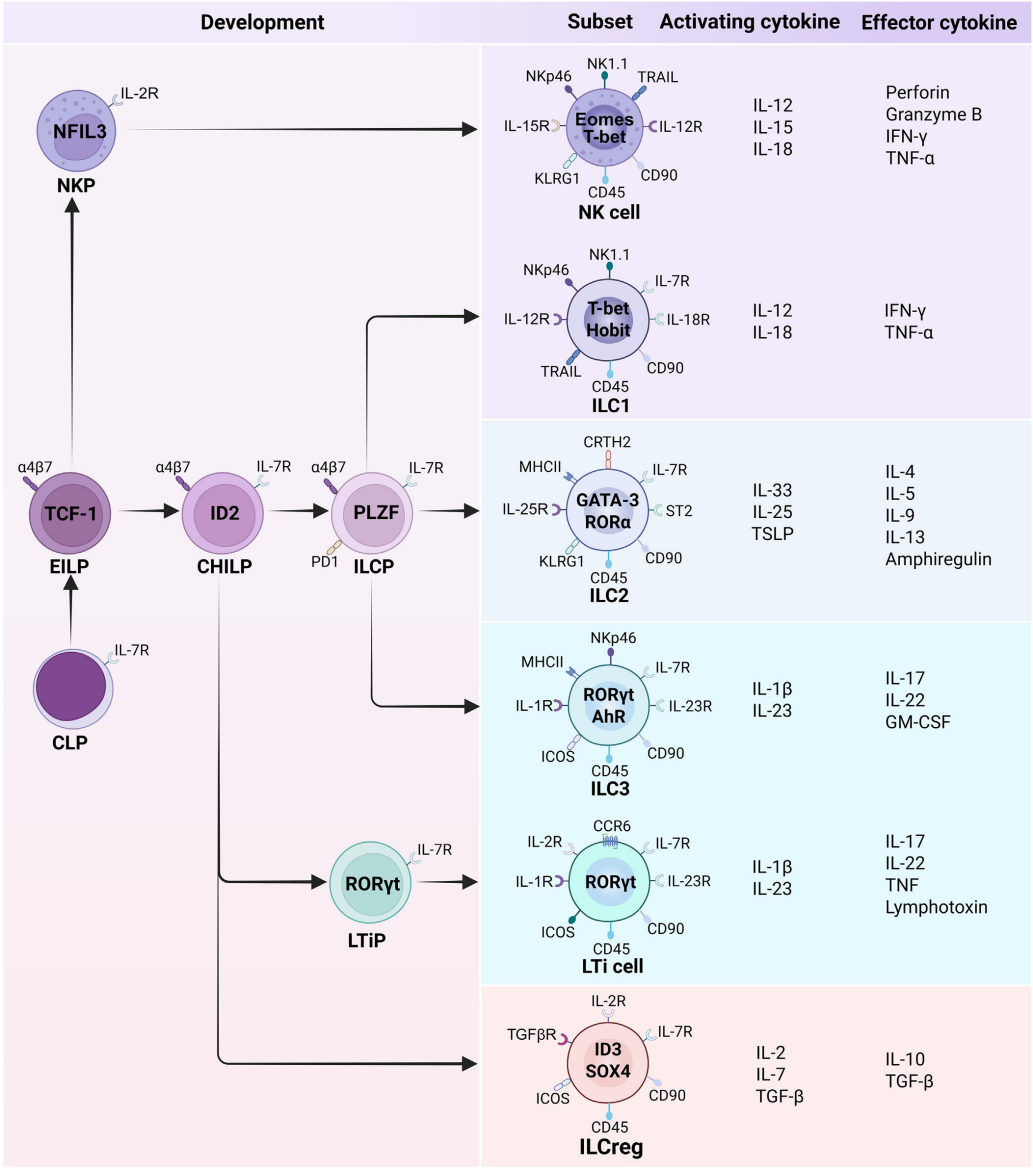
Development and characteristics of ILC subsets. ILCs include Group 1 ILCs (ILC1s and NK cells), Group 2 ILCs (ILC2s), Group 3 ILCs (ILC3s and lymphoid tissue inducer (LTi) cells), and regulatory ILCs (ILCregs). All ILC subsets originate from common lymphoid progenitors (CLPs). Early ILC progenitors (EILPs) can give rise to both NK cell precursors (NKPs) and common helper innate lymphoid progenitors (CHILPs). NKPs further mature into NK cells, while CHILPs differentiate into LTi progenitors (LTiPs), ILC precursors (ILCPs), and ILCregs. LTiPs further develop into LTis, while ILCPs differentiate into ILC1s, ILC2s, and ILC3s. Each subset of ILCs expresses specific transcription factors and produces signature cytokines upon activation (created with BioRender).

ILCs are conventionally classified into five subsets based on the signature cytokine profiles and developmental programs: natural killer (NK) cells, ILC1s, ILC2s, ILC3s, and lymphoid tissue inducer (LTi) cells [1] (Figure 1). All ILC subsets originate from common lymphoid progenitors (CLPs) in the fetal liver or adult bone marrow (BM) [8]. Early ILC progenitors (EILPs), which are downstream of CLPs, can give rise to both NK cell precursors (NKP) and common helper innate lymphoid progenitors (CHILPs) [9]. In response to environmental signals and TFs, NKPs further mature into NK cells, while CHILPs differentiate into LTi progenitors (LTiPs) and ILC precursors (ILCPs). LTiPs further develop into LTis, whereas ILCPs differentiate into ILC1s, ILC2s, or ILC3s [10]. Unlike circulatory NK cells, other ILC populations are predominantly tissue‐resident cells that are maintained in tissues through local self‐renewal [11]. More recently, ILCs have also been described as being present in circulation (cILCs), but only cILC2s have a clear functional correspondence with their tissue‐resident counterparts, whereas cILC1s and cILC3s primarily exist in an immature state [12, 13]. In addition, ILCPs in the blood can respond to signals such as inflammation and be recruited into tissues to support the immune defense of tissue‐resident ILCs [14]. Given that the involvement of NK cells in sepsis has been well elucidated in other reviews [15], this review focuses on non‐NK ILCs in the context of sepsis.

### 2.1 Group 1 ILCs

Group 1 ILCs, which include ILC1s and NK cells, are characterized by their capability to secrete interferon‐γ (IFN‐γ) and transcriptional dependence on the T‐box expressed in T cells (T‐bet). ILC1s and NK cells in mice share surface markers NK1.1 and NKp46 while lacking CD3 [16]. However, unlike NK cells, ILC1s express CD127/IL‐7Rα and do not express the TF Eomes [17]. More recently, Hobit, encoded by Zfp683, has also been proposed to be an essential driver of ILC1 programs [18, 19]. ILC1s were originally considered noncytotoxic because they do not express NK‐like cytolytic effectors, such as perforin and granzyme (Gzm) B [20]. However, recent investigations have identified high expression levels of perforin and Gzm family members, including Gzm A, B, and C, in these cells, which challenges the canonical dichotomy separating ILC1s from NK cells on the basis of their cytotoxic functionality [21, 22]. ILC1s primarily reside in the liver, gut, skin, and tonsils, although they are widely distributed throughout the body and detectable in peripheral blood [16, 18, 19, 23]. During infection, IL‐12 and IL‐18 produced by other cells (e.g., antigen‐presenting cells and stroma cells) promote ILC1 secretion of IFN‐γ and TNF‐α, which are critical proinflammatory cytokines that regulate the activation of immune cells, such as macrophages, dendritic cells (DCs), and CD8^+^ T cells, during sepsis [20, 24, 25, 26]. ILC1s serve as key sources of IFN‐γ and TNF‐α in frontline defense against viral, bacterial, and parasitic pathogens. For example, ILC1‐deficient mice with competent NK cells display impaired IFN‐γ‐mediated recruitment of protective monocytes and defective pathogen clearance during *Toxoplasma gondii* infection [27], which highlights the nonredundant function of ILC1s compared with that of NK cells. However, IFN‐γ and TNF‐α can have pathogenic effects by inducing epithelial cell apoptosis, disrupting the mucosal barrier, and driving detrimental inflammation [28]. Notably, in a clinical cohort study by Carvelli et al., cILC1 counts were markedly greater in patients with septic shock than in healthy volunteers [29]. Conversely, Cruz‐Zárate et al. reported reduced cILC1 and cILC3 counts with a concomitant increase in caspase‐3‐mediated apoptosis in septic patients [30]. Interestingly, ILCs from patients with sepsis exhibited upregulated expression of the activation markers NKp46 and NKp44 [30]. These inconsistent findings could stem from several factors—patient heterogeneity (including variations in the age and genetic background), the timing of sampling (an early hyperinflammatory phase might show ILC expansion, whereas prolonged or severe sepsis could lead to ILC depletion due to apoptosis or exhaustion), and the type of infecting pathogens. These conflicting results underscore the dynamic and context‐dependent nature of ILC responses in sepsis and emphasizes the critical need for well‐designed longitudinal studies that track ILC subsets from early infection through recovery or chronic critical illness to resolve these inconsistencies.

### 2.2 Group 2 ILCs

ILC2s secrete Type 2 cytokines and rely on the expression of GATA binding protein 3 (GATA‐3) and retinoic acid receptor‐related orphan receptor alpha (RORα) for maintenance and survival [8]. ILC2s are widely distributed in barrier tissues (e.g., lungs, skin, intestines) and adipose tissue [31, 32]. Similar to Th2 cells, ILC2s orchestrate allergic inflammation and immunity against helminths. In response to stimuli such as infection and allergens, epithelial or myeloid cells produce IL‐33, IL‐25, and thymic stromal lymphopoietin (TSLP), which in turn serve as alarmins, stimulating ILC2s to secrete IL‐4, IL‐5, IL‐9, and IL‐13 [1, 33]. In Type 2 inflammation, these cytokines drive immunoglobulin class switching in B cells [34], promote eosinophil infiltration, and facilitate degranulation of mast cells [35, 36]. ILC2s can also drive CD4^+^ T cell expansion and Th2 cell differentiation through cytokine secretion and antigen presentation via MHC II molecules [37]. Furthermore, ILC2s secrete amphiregulin (AREG) to facilitate tissue repair and maintain epithelial barrier function through the epidermal growth factor receptor signaling pathway [38]. In addition, ILC2s express various receptors, such as ICOSL, KLRG1, OX40L, and neuromedin U receptor 1 (NMUR1) [38, 39, 40]. These molecules not only regulate the function of ILC2s themselves but also mediate their interplay with immune cells, neurons, and the tissue microenvironment, forming a coregulatory network. Novel evidence has demonstrated that ILC2s can kill bacteria through phagocytic activity and produce extracellular traps via ETosis (extracellular trap formation), similar to NETosis [41, 42]. The diverse properties of ILC2s complicate the elucidation of their roles in sepsis. Notably, a retrospective analysis of patients with sepsis revealed that individuals with Type 1 or Type 17 immunity‐mediated diseases, such as ulcerative colitis and vasculitis, were overrepresented [43]. In contrast, those with preexisting Type 2 immune response‐mediated diseases (e.g., asthma, allergic rhinitis) were significantly underrepresented in the septic cohort, which suggests a potential protective role of Type 2 immune activation in developing sepsis. Further animal studies indicate that ILC2 cytokines balance the necessary but potentially deleterious proinflammatory neutrophilic response in the context of sepsis [43]. On the other hand, Chun et al. reported that ILC2s accumulated at the site of infection in the acute phase of sepsis [44]. ILC2 deficiency or administration of an IL‐33 receptor (ST2)‐blocking antibody decreased levels of the anti‐inflammatory cytokine IL‐10, enhanced bacterial clearance, and improved survival in a cecal ligation and puncture (CLP)‐induced mouse model of sepsis [44]. Similarly, Nascimento et al. demonstrated that ILC2 activation contributed to the development of sepsis‐associated immunosuppression in mice [45]. These findings suggest that the activation of ILC2s during sepsis represents a “trade‐off” between protection from acute hyperinflammation and later manifestation of immunosuppression. Moreover, emerging evidence has revealed that the roles of these cells differ depending on the different tissue microenvironments during sepsis; this will be discussed in detail in subsequent sections.

### 2.3 Group 3 ILCs

Group 3 ILCs exhibit high expression of RORγt and secrete the Type 17 cytokines IL‐17 and/or IL‐22. Group 3 ILCs comprise CCR6^+^NCR^−^ LTi cells or LTi‐like cells, CCR6^−^NCR^−^ ILC3s, and CCR6^−^NCR^+^ ILC3s [46]. LTi cells, which are found only during fetal development, can produce lymphotoxin (LT) to drive secondary lymphoid organogenesis [47]. CCR6^+^NCR^−^ ILC3s are termed LTi‐like cells or “adult LTi cells” since they share similar ontogenetic origins with LTiPs [46]. Two subtypes of CCR6^−^ ILC3s can be distinguished on the basis of the surface expression of NKp44 (NCR1) in human and NKp46 (NCR2) in murine [48]. In contrast to NKp46^−^ ILC3s, NKp46^+^ LC3s in mice also coexpress T‐bet, which is necessary for their development from NCR^−^ ILC3s [49]. Human NKp44^+^ ILC3s also express NKp46 but do not express T‐bet [50]. NCR^−^ ILC3s and LTi cells produce both IL‐22 and IL‐17, whereas NCR^+^ ILC3s produce only IL‐22 [51]. ILC3s are enriched at the mucosal barrier system and predominantly reside in the gut where they are crucial to tissue homeostasis and immune surveillance [52]. In addition to their intestinal predominance, ILC3s also populate lymph nodes, airway, skin, tonsils, and spleen [23]. As the innate counterpart of Th17 cells, ILC3s critically defend against fungal and extracellular bacterial pathogens. In response to metabolites, microbial signals, or myeloid‐derived IL­1β and IL‐23, ILC3s can release IL‐22, IL‐17, GM‐CSF, and other soluble factors, such as heparin‐binding EGF‐like growth factor (HB‐EGF) [9, 53]. ILC3‐derived GM‐CSF, in turn, increases myeloid cell abundance and regulates mononuclear phagocyte (MNP) responses [52]. IL‐22 critically enhances antibacterial host defense by promoting mucus‐associated protein synthesis, antimicrobial peptide production, and epithelial barrier maintenance [54]. IL‐17 synergistically shapes epithelial barrier function with IL‐22 and stimulates the production of chemokines (including CXCL family members) for neutrophil recruitment, which are essential for rapid and effective infection control [55, 56]. ILC3‐derived IL‐22 and IL‐17 have been demonstrated to critically constrain microbial translocation and protect against sepsis [57, 58]. On the other hand, Muir et al. identified ILC3‐derived IL‐17A as a key factor that drives dysregulated inflammatory responses during lipopolysaccharide (LPS)‐induced lung injury and bacterial pneumonia, which suggests its potential role in exacerbating sepsis‐associated organ dysfunction [59]. Notably, both clinical studies by Carvelli et al. and Cruz‐Zárate et al. revealed that patients with sepsis exhibited significantly lower ILC3 numbers in peripheral blood than healthy volunteers [29, 30]. However, evidence for the presence of circulating mature human ILC3s is limited, rendering the clinical significance of their reduction unclear. It remains undetermined whether all human cILC3s are progenitors or whether they represent an intermediate state of tissue‐resident ILC3s.

### 2.4 Regulatory ILCs

In addition to conventional ILCs, a novel regulatory subset of ILCs (termed ILCregs) was identified for the first time in mouse and human intestines [60]. These cells exhibit similarities to regulatory T cells (Tregs), including IL‐10 and TGF‐β production, but lack expression of the signature Treg TF FOXP3 [60]. Additionally, ILCregs express high levels of inhibitor of DNA binding 2 (ID2), which is necessary for the development of all ILCs. Unlike other ILCs, ILCregs uniquely express ID3 and SOX4 and do not express typical TFs, such as RORγt, T‐bet, and GATA‐3. ILCregs produce high levels of IL‐10 to suppress the functional activity of ILC1s and ILC3s, thus resolving intestinal inflammation. Subsequent studies revealed phenotypically similar ILCregs in the renal interstitium that suppress renal inflammation and protect against ischemia–reperfusion injury (IRI) by secreting IL‐10 and TGF‐β [61]. Notably, recent research indicated that IL‐10‐producing ILCs may represent a unique subtype of ILC2s, referred to as ILC2_10_s, which are present in the mouse intestine under both homeostatic and inflammatory conditions [62]. Although these findings raise questions regarding the existence of ILCregs as a distinct subset, the prevailing view in the literature characterizes ILC2_10_s as a functional state transition of ILC2s, in contrast to ILCregs, which are regarded as a separate, stable regulatory lineage [63]. In addition, another ILC population with a distinct phenotype, referred to as follicular regulatory ILCs (ILC_FR_s), was discovered in human lymphoid organs [64]. ILC_FR_s can suppress interactions between germinal center B cells and T follicular helper cells via TGF‐β, thereby reducing IgG production by B cells [64]. Overall, the immunobiology of regulatory ILCs in sepsis is poorly understood; studies are needed to unravel their immunomodulatory networks.

## 3 Plasticity and Heterogeneity of ILCs

**FIGURE 2:**
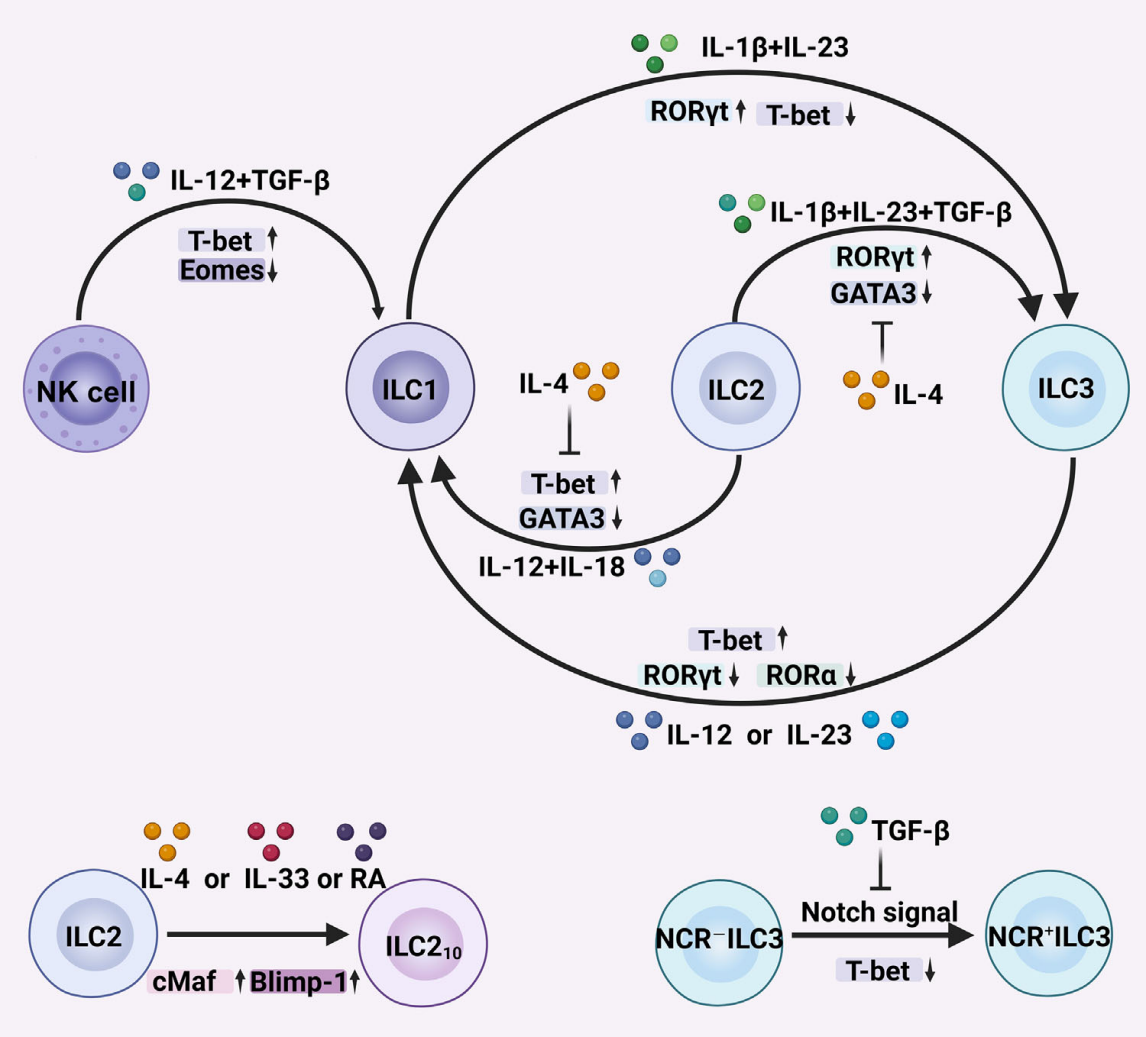
Plasticity of ILCs. ILCs can transdifferentiate into different subtypes in response to environmental cytokines, which is mediated by the upregulation or downregulation of specific transcription factors (denoted by arrows). The conversion of ILC2s into ILC1s or ILC3s can be abrogated by IL‐4. Sustained Notch signals can drive the differentiation of NCR^−^ ILC3s into NCR^+^ ILC3s, whereas TGF‐β inhibits the transition (created with BioRender).In this figure, RA denotes retinoic acid.

Despite lineage‐committing transcriptional programs, ILCs are a heterogeneous family with highly plastic phenotypes and functions that enable transdifferentiation between subsets or acquisition of mixed characteristics beyond their canonical classification [9, 50] (Figure 2). The plasticity of ILCs was first evidenced in mouse and human CCR6^−^ ILC3s, which can transdifferentiate into IFN‐γ‐producing ILC1‐like cells (also termed ex‐ILC3s) [65, 66]. This conversion requires the coordinated downregulation of RORγt and RORα, as well as the upregulation of T‐bet, which can be induced by inflammatory signals such as IL‐12 or IL‐23 [65, 67]. Subsequent studies have reported various transitions between ILC subsets in specific microenvironments, such as infections, inflammatory diseases, and tumors [50, 68]. For example, NK cells, in response to IL‐12 or TGF‐β signaling, can acquire a noncytotoxic ILC1‐like phenotype, characterized by Eomes suppression and T‐bet upregulation [69, 70]; IL‐12 and IL‐15 can drive cytotoxic NK cell differentiation in ILC3s [71]. In addition, sustained Notch signaling can induce the transdifferentiation of NCR^−^ ILC3s into NCR^+^ ILC3s by downregulating T‐bet expression, whereas TGF‐β inhibits this process [72]. Emerging evidence has demonstrated the phenotypic plasticity of intestinal ILCs in a *Pseudomonas aeruginosa* pneumonia‐induced mouse model of sepsis, characterized by the decreased number of phenotypic NKp46^+^ ILC3s and a corresponding increase of ILC1s [73]. These findings suggest that sepsis‐induced gut inflammation establishes a cytokine milieu that is capable of triggering the transdifferentiation of ILC3s into ILC1s. However, the exact mechanism and biological impact of ILC3 plasticity in the context of sepsis remain unresolved. Although the conversion of ILC3s to ILC1s can drive detrimental inflammation in some conditions, such as colitis, it may also protect against sepsis by bolstering antimicrobial immunity and inducing mucus production to safeguard the epithelial barrier through the secretion of IFN‐γ [74]. Notably, the conversion of ILC3s to ILC1s is invertible, as IL‐1β and IL‐23 can induce the reconversion of human ILC1s to IL‐22‐secreting NKp44^+^ ILC3s, and this transformation can be accelerated by retinoic acid [65].

ILC2s also exhibit remarkable plasticity and can dynamically shift between protective and pathogenic phenotypes in response to microenvironment cues. The transdifferentiation of ILC2s to IFN‐γ‐secreting ILC1s has been demonstrated in murine lungs infected with influenza viruses [75]. Stimulation with IL‐12 and IL‐18 can drive this conversion through GATA‐3 suppression and T‐bet induction. ILC2s in the mouse lung transdifferentiate into IL‐17‐producing ILC3s in response to IL‐25, and these IL‐25‐responsive ILC2s, termed inflammatory ILC2s (iILC2s), protect against helminth infection [76]. Unlike tissue‐resident natural ILC2s (nILC2s), which respond primarily to IL‐33, iILC2s can produce both IL‐13 and IL‐17 and migrate via circulation to protect distal tissues. Compared with nILC2s, iILC2s express RORγt and abundant levels of KLRG1 but lack ST2 expression [76]. Further studies have demonstrated that human ILC2s can also convert to iILC2s upon stimulation with IL‐1β, IL‐23, and TGF‐β, which are released by the epithelium infected with *Staphylococcus aureus* or *P. aeruginosa* [77]. IL‐4 can inhibit the conversion of ILC2s to ILC1s or ILC3s. Several groups have also reported that IL‐10‐producing ILC2s (ILC2_10_s) reduce local inflammation [78, 79, 80]. IL‐10 production in ILC2_10_s can be driven by IL‐33, IL‐4, or retinoic acid and requires the upregulation of cMaf and Blimp‐1 [79]. Overall, these findings highlight the plasticity and environmental heterogeneity of ILCs, which makes it more challenging to clarify their mechanistic involvement in sepsis. Strategies that target ILC plasticity may ameliorate immune disorders by selectively enhancing pathogen clearance while attenuating hyperinflammatory responses in sepsis.

## 4 Role of ILCs in Homeostasis

In addition to their recognized roles in pathogen clearance and inflammation, ILCs are central regulators of physiological homeostasis. Their strategic positioning across barrier tissues, metabolic organs, and lymphoid structures enables them to function as key integrators of environmental, microbial, and neuronal signals. This section examines the multifaceted homeostatic functions of ILCs, highlighting their critical roles in three interconnected pillars of health: immune defense and tolerance, tissue integrity and repair, and metabolic regulation. Understanding these roles reveals that ILCs are indispensable for daily physiological maintenance and that their dysregulation is a critical nexus to disease pathogenesis.

### 4.1 Immunological Defense and Tolerance

The body is continuously exposed to potential pathogens, and defense against these threats is essential for the maintenance of tissue homeostasis. Owing to the wide distribution of heterogeneous ILCs across various tissues and organs in the body, these cells play a crucial role in the immune response against various pathogens, including viruses, bacteria, fungi, and intracellular and extracellular parasites.

IFN‐γ and TNF‐α, the signature cytokines of ILC1s and NK cells, are recognized for their antiviral and immunomodulatory functions [81]. As tissue‐resident cells, ILC1s rapidly congregate at infection sites to enhance local innate defense and confer early host protection against various infections by directly killing pathogens and activating other immune cells. ZFP683 (ZNF683 in humans) is critical for the tissue residency and functional maturation of ILC1s [18]. In Hobit‐deficient mice (a model lacking hepatic ILC1s but with preserved conventional NK cells), ILC1s critically limited the liver viral load during mCMV infection, independent of conventional NK cells and adaptive immunity [82]. Type 1 conventional DCs (cDC1s) can produce interleukin (IL)‐12 to promote IFN‐γ production by ILC1s in a STAT4‐dependent manner [82]. This ILC1‐derived IFN‐γ, in turn, is indispensable for inducing the expression of IRF8 (an essential TF for cDC1s) in cDC1s, thereby upregulating their proinflammatory activity at the infection site [83]. These findings indicate that ILC1s engage in extensive interactions with other immune cells to enhance immune defense and maintain homeostasis. Under homeostatic conditions (in the absence of infections), ILC1 can secrete IFN‐γ to maintain a basal antiviral state and provide barrier‐tissue protection through immune surveillance [84]. In addition, a population of Gzm expressing, IL‐7R^−^ ILC1s with cytotoxic activity under steady‐state conditions has recently been identified [85]. Unlike NK cells, whose cytotoxicity is dependent on exogenous stimulation, those cytotoxic ILC1s are proposed to function as critical sentinels in steady‐state immune surveillance against infections and tumors [85].

ILC2s are critical for host resistance against helminth infections. Upon helminth infection, intestinal tuft cells produce IL‐25, which activates ILC2s to release IL‐13. This cytokine orchestrates worm expulsion by inducing smooth muscle contraction, mucus production from goblet cells, and recruitment of macrophages and eosinophils [33]. Furthermore, IL‐13 can act on stem cells to promote their differentiation into tuft and goblet cells, reinforcing the epithelial barrier for parasite clearance. Interestingly, the IL‐25/IL‐13 axis is also active during homeostasis. This ILC2‐mediated immunity drives adaptive small‐intestinal lengthening and remodeling during the development of the gut [86]. Constitutive IL‐25 production by tuft cells maintains basal ILC2 activation, while tuft cell‐intrinsic IL‐17RB (IL‐25 receptor) expression homeostatically regulates this circuit by controlling local IL‐25 bioavailability [87]. In healthy skin, homeostatic IL‐13, which is secreted by dermal ILC2s, directs DC2 differentiation to promote TH2 and inhibit TH17 cell polarization, thereby controlling the balance between Type 2 and Type 3 immunity [88]. In the lung, the homeostasis and activation of tissue‐resident ILC2s are primarily regulated by IL‐33, while circulating iILC2s that produce IL‐17 to combat infection are thought to originate from intestinal ILC2 populations [89, 90]. Furthermore, during helminth infection, ILC2s at mucosal barriers synthesize acetylcholine to amplify their expansion and protective immunity [91]. In contrast, interactions with neuronal signals can suppress excessive ILC2 activation. For example, ILC2s express the β_2_‐adrenergic receptor and colocalize with adrenergic neurons in the intestine, which cell‐intrinsically suppresses ILC2 responses by inhibiting proliferation and effector functions [92]. Similarly, dopamine has been shown to suppress excessive Type 2 immune responses by inhibiting the mitochondrial oxidative phosphorylation pathway in lung ILC2s [93]. Therefore, in addition to their well‐established role in orchestrating antihelminth immunity, ILC2s function as central coordinators in maintaining tissue homeostasis, a process fine‐tuned by neuronal circuits to ensure a balanced immune response.

ILC3s are key regulators of antibacterial responses and immune tolerance. As previously described, ILC3s combat bacterial pathogens by enhancing barrier defenses and modulating other immune cells via cytokine secretion. ILC3s contribute to pathogen tolerance early in infection by initiating tissue‐protective immunity. Although compensatory T‐cell responses can maintain survival in ILC3‐deficient hosts under low‐dose infection, ILC3s play a nonredundant and indispensable role in survival when confronted with a high infectious dose [94]. Recently, ILC3‐derived colony stimulating factor 2 (CSF2) has also been identified as a key regulator of myeloid cell development and maintenance in multiple organs (including the gut, liver, and spleen) under homeostasis [95]. Furthermore, Jarrade et al. demonstrated that intestinal villus ILC3s, which are largely immotile at steady state, can acquire a migratory patrolling attribute and increase cytokine production upon inflammatory challenge [96]. Thus, ILC3s sustain a state of low‐level immune surveillance under homeostasis, ensuring a rapid response to tissue damage or pathogen invasion. Another critical physiological function of ILC3s is to promote immune tolerance. The ability to tolerate harmless dietary antigens and commensal gut microbiota while maintaining defense against pathogenic pathogens is essential for gastrointestinal homeostasis. Under homeostatic conditions, intestinal ILC3s constitutively express MHC II but lack expression of costimulatory molecules [97]. Owing to the absence of costimulation, antigen presentation by ILC3s induces the apoptosis of highly reactive CD4^+^ T cell clones, thereby suppressing the overactivation of proinflammatory T cells while supporting microbiota‐specific RORγt^+^ Tregs [98]. Studies have further revealed that the TF BATF and IFN regulatory factor 4 (IRF4) epigenetically regulate MHC II expression on ILC3s and control their functional plasticity [99, 100]. BATF acts downstream of IRF4 to increase MHC II expression, forming a coherent transcriptional hierarchy initiated by the binding of IRF4 to the *Batf* locus [99]. BATF ablation drives the expansion of ILC1‐like ILC3s and promotes IFN‐γ production, thereby aggravating intestinal inflammation [100]. Beyond direct contact, ILC3s also maintain the pool of Tregs and support oral tolerance by secreting IL‐2 [101]. This IL‐2‐dependent mechanism is orchestrated by gut commensal microbes, which activate macrophages to produce IL‐1β, thereby stimulating ILC3s to release IL‐2 [101]. In summary, ILC3s are pivotal for maintaining intestinal homeostasis by integrating microbial and environmental cues to orchestrate a balanced immune response. This is achieved through their critical roles in supporting immune tolerance, defending against pathogens, and controlling inflammation.

### 4.2 Tissue Integrity and Repair

LTi cells are indispensable for the formation of secondary lymphoid tissues, such as lymph nodes and the spleen. During embryogenesis, LTi cells express LTα1β2, which interacts with LTβR on mesenchymal stromal cells [102, 103]. This interaction triggers the release of chemokines and adhesion molecules, facilitating the recruitment of T and B cells and ultimately leading to lymph node organogenesis. Consequently, RORγt‐deficient mice, which lack ILC3s (including LTi cells), fail to develop these lymphoid structures. Intriguingly, Stehle et al. recently reported that concomitant deletion of T‐bet rescues lymph node formation in RORγt‐deficient mice [104]. The study demonstrates that the relative expression levels of RORγt and T‐bet act as critical determinants in the differentiation of ILCP, thereby controlling the ILC3/ILC1 balance during embryogenesis [104]. The proportion of LTi cells decreases significantly after birth, a process that can be partially attributed to enhanced Notch signaling in adult ILCPs [105]. This heightened signaling orchestrates a developmental shift from a fetal LTi‐dominant state to an adult state dominated by non‐LTi ILCs, thereby meeting the body's evolving requirements for distinct ILC subsets at different life stages [105]. In addition to their role in the formation of secondary lymphoid structures, ILC3s also significantly contribute to barrier integrity and tissue repair. For instance, intestinal epithelial cells, which are composed of multiple specialized cell types, such as Paneth cells, goblet cells, stem cells, and enterocytes, collectively form a monolayer barrier crucial for intestinal homeostasis and defense [52]. At steady state, ILC3s serve as a primary source of IL‐22 in various barrier tissues including the intestine [52, 94]. IL‐22 upregulates epithelial expression of tight junction proteins, induces Paneth cells to secrete antimicrobial peptides (e.g., RegIIIγ and defensins) and promotes the differentiation of goblet cells along with thickening of the mucus layer, thereby enhancing the physicochemical barrier [94, 106]. ILC3s may also help support intestinal barrier integrity through their migratory patrolling behavior, which facilitates the diffusion of IL‐22 to prevent epithelial cell death [96]. Furthermore, IL‐22 upregulates IL‐18 expression via the STAT3 signaling pathway [107]. IL‐18, in turn, facilitates the expansion of Lgr5^+^ stem cells to promote tissue repair [107]. The production of IL‐22 is actively regulated by the gut microbiota and diet signals. In turn, ILC3‐derived IL‐22 plays a crucial role in maintaining microbial homeostasis. For instance, microbiota‐derived metabolites can activate the aryl hydrocarbon receptor (AhR) pathway to stimulate ILC3s, preventing dysbiosis and supporting barrier function by modulating gut metabolism in an IL‐22‐dependent manner [108, 109]. Food‐induced expression of the neuropeptide vasoactive intestinal peptide (VIP) can significantly increase IL‐22 production and epithelial barrier function by activating VIP receptor 2, which is highly expressed on gut ILC3s [110, 111]. Support of the intestinal barrier by ILC3s also involves non‐IL‐22 mechanisms, such as promoting tissue regeneration through Hippo–Yap1 signaling activation in crypt cells and providing protection from inflammation‐induced epithelial cell death via HB‐EGF production [53, 112].

ILC2s produce AREG, a ligand of the EGF receptor that modulates cell proliferation or differentiation and promotes tissue repair through binding to the EGF receptor on various cell types. IL‐33‐stimulated ILC2s secrete AREG to mediate tissue repair and promote wound healing following viral infection and inflammation [113, 114]. For instance, in a model of cerebral ischemia, ILC2s accumulated in the ischemic region, where they secreted AREG to augment the proliferation of neural stem and progenitor cells, thereby improving functional recovery [115]. Intriguingly, in addition to secreting AREG to aid in cardiac repair post myocardial injury, IL‐33‐stimulated ILC2s alleviate fibrosis through a multifaceted mechanism involving EGFR signaling, IL‐13‐induced M2 macrophage activation, and BMP‐7‐dependent antifibrotic effects [116]. Russi et al. discovered that the plasticity between protective nILC2s and iILC2s, regulated by an intrinsic IL‐13/IL‐4Rα/STAT6 circuit, affects epithelial repair in biliary atresia by controlling the production of AREG [117]. However, overactivation of AREG‐mediated repair response can also contribute to pathological fibrosis such as Hermansky–Pudlak syndrome [118, 119]. Therefore, the repair function of ILC2s mediated by AREG is ambivalent, and its outcome depends on the precise regulation of the tissue microenvironment to prevent the transformation from physiological repair to pathological fibrosis.

### 4.3 Metabolic Regulation

In recent years, ILCs, particularly ILC2s in adipose tissue, have been identified as crucial regulators of metabolic homeostasis. ILC2s maintain metabolic balance through multiple mechanisms, including sustaining Type 2 immunity and promoting the process known as adipose tissue beiging [120, 121]. Adipose tissue is primarily classified into white adipose tissue (WAT), which stores excess energy as triglycerides, and brown adipose tissue (BAT). Brown adipocytes express uncoupling protein 1 (UCP1), which uncouples mitochondrial respiration from ATP synthesis, dissipating energy as heat. ILC2s contribute to energy expenditure by producing methionine–enkephalin peptides, which upregulate UCP1 expression in white adipocytes and promote their differentiation into beige adipocytes [122]. Furthermore, upon stimulation by IL‐33, ILC2s secrete Type 2 cytokines such as IL‐4 and IL‐13. These cytokines can directly stimulate the proliferation of adipocyte precursors expressing IL‐4Rα and drive their differentiation toward the beige adipocyte lineage with BAT‐like functionality [123]. Additionally, ILC2‐derived IL‐5 recruits eosinophils, which in turn secrete IL‐4 to help maintain a population of M2 macrophages [124]. These cells collectively support a Type 2 immune environment and inhibit inflammation in adipose tissue. The observed reduction in ILC2 responses within WAT is a phenotype consistently associated with obesity in both human subjects and mouse model tissue [122]. The impaired ILC2 function subsequently results in decreased numbers of eosinophils and M2 macrophages, thereby promoting pathological adipose inflammation and insulin resistance following high‐fat diet (HFD) feeding [124]. Intriguingly, recent research has identified a neuro‐mesenchymal unit that translates sympathetic signals from brain circuits into ILC2 regulation in adipose tissue via GDNF signaling, directly controlling host metabolism and obesity propensity through a brain–adipose axis [125].

In addition to their role in adipose tissue, ILC2s have recently been identified as direct regulators of glucose metabolism in multiple organs. In hepatocytes, IL‐13 produced by liver‐resident ILC2s mediates the STAT3 signaling‐dependent downregulation of the expression of the gluconeogenic enzyme glucose‐6‐phosphatase expression [126]. This leads to suppressed hepatic gluconeogenesis and a consequent decrease in blood glucose levels [126]. In the pancreatic islets, IL‐33 released by stromal cells activates islet‐resident ILC2s to produce IL‐13 and GM‐CSF, which in turn promote the release of retinoic acid by myeloid cells; retinoic acid then acts on β‐cells to enhance insulin secretion, a pathway crucial for maintaining islet function and preventing Type 2 diabetes (T2D) [127]. Furthermore, a groundbreaking discovery revealed a dynamic, interorgan circuit in which ILC2s migrate to regulate systemic glucose [128]. During fasting, adrenergic neuronal signaling promotes the migration of ILC2s from the intestine to the pancreas. These pancreatic ILC2s then produce Type 2 cytokines, which stimulate glucagon production [128]. This process triggers endogenous glucose production in the liver, highlighting a novel neuro‐immune–endocrine axis for maintaining energy homeostasis during low‐energy states. Thus, ILC2s emerge as pivotal integrators of metabolic homeostasis, capable of performing diverse and even antagonistic functions—such as both suppressing and promoting gluconeogenesis—in a tissue‐specific and state‐dependent manner to ensure systemic metabolic balance.

Emerging evidence indicates that ILC3s also possess metabolic regulatory functions. ILC3s help maintain commensal microbiota homeostasis by mediating IL‐22‐dependent regulation of intestinal galactosylation in mice [109]. ILC3 deficiency results in aberrant galactosylation, leading to microbial dysbiosis and increased host susceptibility to enteric pathogens [109]. Furthermore, while IL‐22 has been confirmed to regulate glucose and lipid metabolism in multiple organs to maintain homeostasis, future studies are needed to delineate the specific contributions of ILC3s to these metabolic functions [129].

In summary, ILCs are central regulators of physiological homeostasis, immune coordination, tissue repair, and metabolism. When the delicate balance of immune homeostasis is disrupted, the mechanisms that ILCs employ for tissue protection and metabolic regulation can be subverted, contributing to disease pathogenesis. This paradigm provides an essential baseline for deciphering their complex, often dualistic roles in pathological conditions such as sepsis.

## 5 ILCs in Diseases: A Paradigm of Sepsis

Sepsis is a serious medical condition that is triggered by dysregulated host responses to infection [5]. The pathophysiology of sepsis is complex and involves inflammatory imbalance, coagulation disorders, and metabolic reprogramming [130]. Its hallmark features of profound immune dysregulation and multiorgan dysfunction, serve as a paramount paradigm for dissecting the complex and context‐dependent roles of ILCs in diseases. In this chapter, we first elucidate the intricate interactions between ILCs and key immune populations in the septic milieu, unraveling the cellular networks that dictate disease progression. Subsequently, we focus on the tissue‐specific roles of ILCs, examining how they orchestrate or mitigate organ injury at critical sites such as the lungs, gut, and heart during sepsis. Through this focused analysis, we aim to illustrate how sepsis provides a powerful lens through which to view the dualistic nature of ILCs—as both guardians of immunity and potential drivers of pathology.

### 5.1 Interaction of ILCs with Immune Cells in Sepsis

**FIGURE 3:**
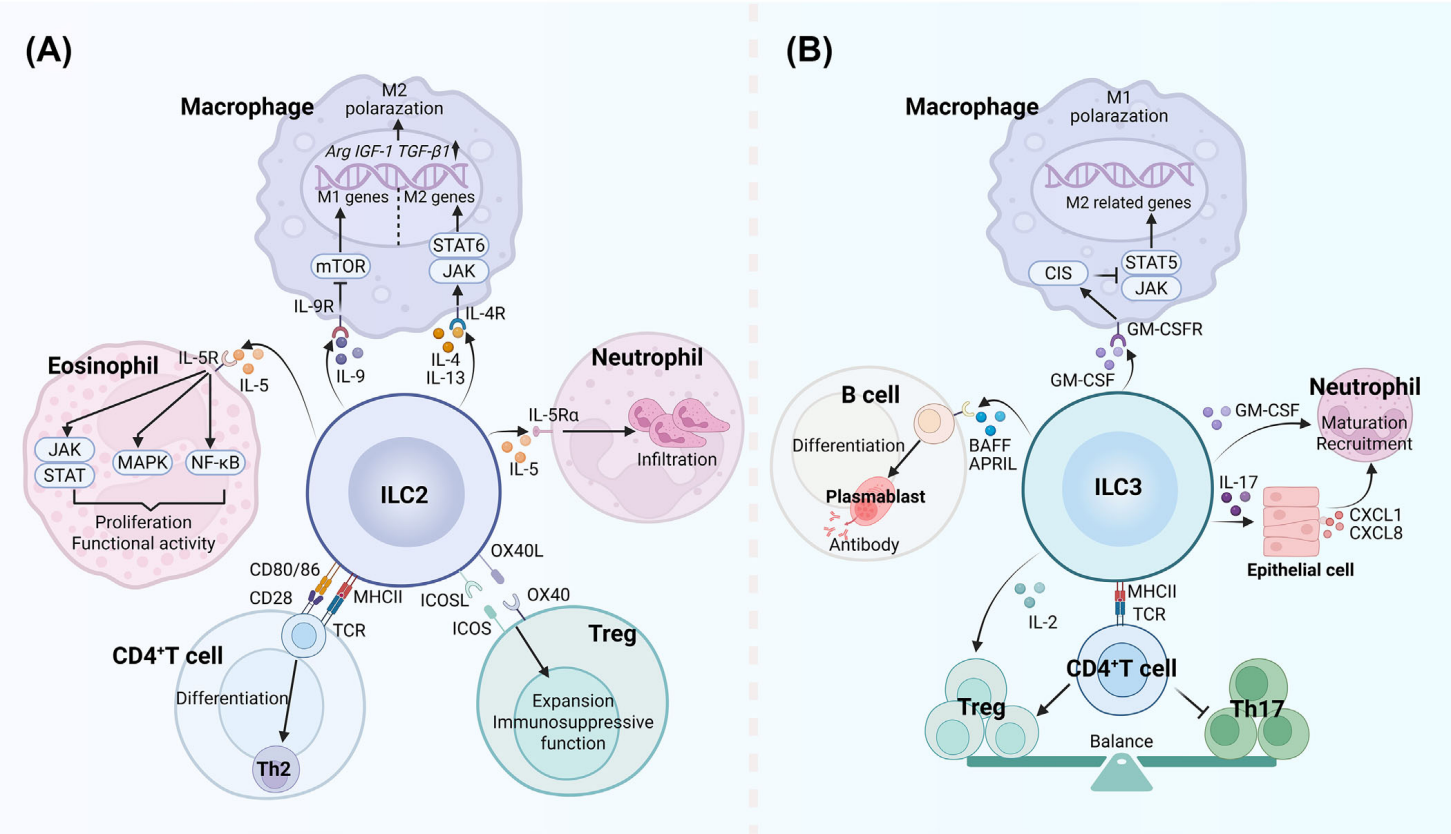
ILCs regulate the function of immune cells in sepsis. (A) ILC2‐derived IL‐9 suppresses mTOR signaling to inhibit M1 macrophage polarization, while IL‐4 and IL‐13 activate the JAK–STAT6 pathway to promote M2 polarization. IL‐5 activates eosinophils via the JAK–STAT, MAPK, and NF‐κB pathways and recruits IL‐5Rα^+^ neutrophils. Direct contact between MHC–TCR and costimulatory molecules promotes Th2 differentiation, whereas OX40L–OX40/ICOSL–ICOS interactions enhance Treg expansion and function. (B) ILC3‐derived GM‐CSF potentially suppresses the JAK–STAT5 signaling pathway via cytokine‐inducible SH2‐containing (CIS) proteins activation, promoting M1 macrophage polarization. Both GM‐CSF and IL‐17 enhance neutrophil maturation and recruitment via direct or indirect mechanisms, such as by inducing chemokine expression in epithelial cells. ILC3‐secreted IL‐2 promotes Treg survival. Through MHC–TCR contact, ILC3s suppress the overactivation of Th17 cells while supporting Tregs, thereby maintaining homeostasis. ILC3s also facilitate B cell activation, plasmablast differentiation, and antibody production through the expression of BAFF and APRIL (created with BioRender).

The immune response in sepsis is a highly complex network that involves the dynamic interplay between innate and adaptive immune cells. As versatile innate sentinels, ILCs orchestrate immune responses not only through cytokine production but also via bidirectional interactions with diverse immune populations. Here, we delineate how ILCs regulate key immune cells (Figure 3), providing novel insights into sepsis immunopathology.

#### 5.1.1 Macrophages

As central components of innate immunity, macrophages serve as first responders to pathogens and dynamically regulate tissue homeostasis. In sepsis, the functional polarization of macrophages into M1 (proinflammatory) and M2 (anti‐inflammatory) subtypes reflects a double‐edged sword: M1 macrophages drive pathogen clearance through proinflammatory cytokine (e.g., IL‐1β, TNF‐α, and IL‐6) production and antigen presentation, but their sustained activation exacerbates tissue damage and organ dysfunction [131]. Conversely, M2 macrophages mitigate inflammation and promote tissue repair via IL‐10 and TGF‐β, but their overabundance risks immunosuppression, enables secondary infections, and delays recovery. An imbalance of M1/M2 macrophages can exacerbate sepsis pathogenesis, and emerging evidence reveals that ILCs dynamically modulate macrophage polarization. As a key effector of ILC1 and NK cells, IFN‐γ drives M1 macrophage polarization by activating JAK–STAT1 signaling [132]. In contrast, ILC2‐derived IL‐4 and IL‐13 are canonical mediators that facilitate M2 polarization. IL‐4 and IL‐13 share the coreceptor IL‐4 receptor alpha (IL‐4Rα) and can activate JAK1–STAT6 signaling, which promotes the expression of M2‐related genes. In the CLP sepsis mouse models, IL‐33–ST2 signaling drives ILC2 expansion and IL‐4/IL‐13 secretion, thus promoting M2 polarization [45]. The upregulation of M2‐related genes, including *Arg1*, *IGF‐1*, and *TGF‐β1*, promotes tissue repair, whereas IL‐10 produced by M2 macrophages contributes to immunosuppression in sepsis‐surviving mice. Similarly, in burned mice, intestinal ILC2s in the lamina propria were shown to inhibit M1 polarization and impair macrophage‐mediated antimicrobial defense against sepsis originating from bacterial translocation [133]. Further studies indicate that other Type 2 cytokines, including IL‐5 and IL‐9, also regulate macrophage polarization in sepsis. For example, IL‐5 depletion enhances LPS‐triggered M1 macrophage polarization by activating the NF‐κB–p65 pathway [134], whereas ILC2‐derived IL‐9 inhibits mTOR signaling to suppress macrophage apoptosis and M1 polarization [135].

ILC3s also play pivotal roles in regulating macrophage polarization. During intestinal infection or inflammation, ILC3‐derived GM‐CSF promotes M1 macrophage differentiation [136]. However, GM‐CSF can also drive an immunosuppressive M2‐like phenotype under certain conditions, which highlights its context‐dependent effector mechanism. Further studies demonstrated that cytokine‐inducible SH2‐containing proteins have a marked effect on GM‐CSF‐induced macrophage polarization, since their absence results in hyperactivation of the JAK–STAT5 pathway and promotes M2‐like macrophage development [137]. Finally, ILCregs have emerged as potential regulators of macrophage polarization in the context of sepsis. ILCregs in the kidney were found to suppress effector functions of M1 macrophages and promote the switch of macrophages toward the M2 phenotype by secreting IL‐10 and TGF‐β [61]. Although these studies provide insight into the regulation of macrophages by specific cytokines, further research is needed to clarify the spatiotemporal expression patterns of ILC‐derived cytokines in sepsis and whether these cytokines act synergistically or antagonistically.

#### 5.1.2 Neutrophils

Neutrophils function as critical defenders against infection in sepsis via phagocytosis, degranulation, and neutrophil extracellular trap formation to kill pathogens directly. However, their dysregulation also drives uncontrolled inflammation and tissue damage, which exacerbates organ dysfunction. While ILC2s predominantly recruit eosinophils through Type 2 cytokines, studies have shown that they also have a regulatory effect on neutrophils. Krishack et al. revealed that intratracheal IL‐33 pretreatment induces ILC2s to secrete IL‐5 and IL‐13, driving eosinophil expansion while suppressing bacteremia‐induced neutrophilia during sepsis [43]. However, it is also possible that decreased neutrophil infiltration is caused by eosinophil‐ or ILC2‐mediated decreases in the bacterial load rather than a direct effect on neutrophils. Conversely, recent studies revealed that neutrophils in the blood and lungs express the IL‐5 receptor alpha chain (IL‐5Rα, CD125) in specific contexts and that IL‐5 can induce neutrophil infiltration. For example, Xu et al. demonstrated that pulmonary neutrophils exhibit high expression levels of IL‐5Rα in the context of sepsis [138]. IL‐5 produced by ILC2s mediates neutrophil activation and recruitment into the lungs, which contributes to early inflammation‐associated lung injury during sepsis [138]. Moreover, ILC2s may also regulate neutrophils in sepsis in a cytokine‐independent manner. Emerging evidence reveals that ILC2s promote neutrophil accumulation in the lungs by releasing high mobility group box‐1, which is a DAMP loaded onto extracellular lipid droplets, inducing CXCL2 production by neutrophils to increase their recruitment [139]. In contrast, ILC1s employ a distinct, IFN‐γ‐dependent mechanism to modulate inflammation in sepsis [140]. During early‐stage sepsis, ILC1‐derived IFN‐γ facilitates the egress of anti‐inflammatory CD49d^−^ immature neutrophils (imNeus) from the BM by downregulating CXCR4 expression [140]. Once recruited, these imNeus produce substantial IL‐10, thereby dampening the systemic cytokine storm and mitigating tissue inflammation.

ILC3 regulation of neutrophil homeostasis through IL‐17 has been shown to be important in the prevention of neonatal late‐onset sepsis [57]. Specifically, ILC3s secrete IL‐17 to induce granulocyte colony‐stimulating factor (G‐CSF)‐mediated emergency granulopoiesis in the BM and promote neutrophil recruitment to combat bacterial invasion [57]. Similarly, ILC3s, especially NKp46^+^ ILC3s, enhance early neutrophil‐mediated antimicrobial immunity by secreting GM‐CSF, which promotes neutrophil maturation in the BM [141]. ILC3s also produce chemokines such as CXCL2, which directly recruit neutrophils by binding to CXCR2 [141]. Conversely, another study demonstrated that ILC3‐derived IL‐22 suppresses neutrophil accumulation and downregulates the expression of S100A8/S100A9, which is associated with neutrophilic inflammation [142]. Notably, IL‐22 produced by ILC3s has also been reported to promote neutrophil recruitment and exacerbate tissue injury in several settings, suggesting its highly context‐dependent function [143].

#### 5.1.3 T Cells

During immune responses, CD4^+^ T cells differentiate into functionally distinct subsets (e.g., Th1, Th2, Th17, and Treg cells) via TCR signaling, and each of these subsets has specialized functions. Accumulating evidence highlights the crucial role of the dynamic balance between Th subsets and Tregs in sepsis outcomes, and the dysregulation of proinflammatory Th17 cells and immunosuppressive Tregs has emerged as a critical driver of sepsis pathophysiology via the induction of immune disorders [144]. Recent studies revealed that ILCs dynamically influence this balance. As mentioned previously, ILC2s contribute to persistent immunosuppression in sepsis survivors through indirect interactions with Tregs in a CLP‐induced mouse model [45]. Specifically, IL‐33 that is released from damaged tissues induces ILC2 expansion and IL‐4/IL‐13 production, driving the polarization of M2 macrophages, which in turn produce IL‐10 to amplify Treg expansion and render sepsis‐survivors more susceptible to secondary infection. IL‐33 can also induce ILC2‐mediated engagement with Tregs through OX40L–OX40 and ICOSL–ICOS interactions, thereby promoting Treg expansion and enhancing the immunosuppressive functions of these cells [39, 145]. Notably, ILC2s can also amplify the Th17‐like response. A recent study revealed that sepsis activates ILC2s via the NMUR1 signaling pathway, inducing IL‐9 secretion [146]. This ILC2‐derived IL‐9 promotes pulmonary γδ T cell proliferation and IL‐17A production through IL‐9 receptor signaling [146]. The NMU administration increased pulmonary ILC2/γδ T cell abundance and improved sepsis survival, suggesting the involvement of an uncharacterized immunomodulatory pathway.

ILC3s are essential for maintaining the Treg/Th17 balance in a physiological state. GM‐CSF and IL‐2 produced by ILC3s are vital for maintaining Treg and immunologic homeostasis, and their reduction is correlated with decreased Treg cell numbers [101, 147]. Furthermore, ILC3s mediate antigen presentation via MHC II molecules and orchestrate inflammatory responses through interplay with T cells. Under homeostatic conditions, intestinal ILC3s constitutively express MHC II but lack expression of costimulatory molecules [97]. Owing to the absence of costimulation, antigen presentation by ILC3s induces the apoptosis of highly reactive CD4^+^ T cell clones, thereby suppressing the overactivation of Th1/Th17 cells while supporting microbiota‐specific RORγt^+^ Tregs [98]. The impairment of the ILC3‐mediated balance between Th17 cells and Tregs could exacerbate pathogenic inflammation. Notably, upon activation by IL‐1β, peripheral NCR^−^ ILC3s upregulate MHC II and express costimulatory molecules, including CD40, CD80, and CD86 [148]. These activated ILC3s promote antigen‐specific T cell priming and expansion [148]. Collectively, these findings reveal that ILC subsets differentially regulate the Treg/Th17 balance through context‐dependent cytokine networks and cell‒cell interactions. Targeting these immunomodulatory pathways may offer therapeutic leverage to either amplify or suppress T cell responses in sepsis.

#### 5.1.4 B Cells

B cells are key components of adaptive immunity and are responsible for antibody production, antigen presentation, and cytokine secretion. Decreased B cell numbers and impaired function are associated with immunosuppression and poor prognosis in patients with sepsis [149]. ILCs serve as the key coordinators of B cell function. Intestinal ILC3s secrete LT‐α1β2 and LT‐α3 to drive IgA production by B cells [150], which limits pathogen translocation and sepsis‐associated bacteremia. In the spleen, RORγt^+^ ILCs promote marginal zone (MZ) B cell activation, plasmablast differentiation, and antibody production through the expression of BAFF, APRIL, CD40L, and Notch ligands [151]. Splenic MZ B cells serve as frontline defenders during sepsis by rapidly producing antibodies against blood‐borne pathogens and producing cytokines (e.g., TNF‐α and IL‐10) to modulate inflammation. Splenic RORγt^+^ ILCs can also further enhance MZ B cell responses by activating perifollicular neutrophils via GM‐CSF [151]. Furthermore, ILC3s in peripheral blood promote naive B cell survival, expansion, and differentiation into IL‐10‐producing PD‐L1^+^ regulatory B cells through coordinated CD40L and BAFF signals [152]. In addition, ILC2‐derived IL‐5 is necessary for the development and function of B1 cells [153]. ILC2‐deficient mice present reduced numbers of peritoneal B1 cells with phosphatidylcholine‐specific BCR rearrangements and produce less IgM/IgE in Type 2 inflammation [153]. B1 cells play crucial roles in host defense and inflammatory regulation through their dual capacity to secrete IgM and IL‐10. Notably, B1 cells in the peritoneal cavity are significantly decreased in sepsis, whereas maintaining peritoneal B1 cell homeostasis is critical for mitigating inflammation and improving survival in a CLP mouse model [154]. Thus, further investigations are warranted to elucidate whether sepsis affects the function of ILCs, thereby disrupting their supportive role in B‐cell proliferation or antibody production.

### 5.2 Tissue‐Specific Roles of ILCs in Sepsis

The tissue‐specific distribution and functional adaptation of ILCs highlight their pivotal roles in orchestrating immune responses that are tailored to local microenvironments during sepsis. Emerging evidence reveals that distinct ILC subsets exhibit tissue‐specific regulatory dynamics, contributing to both protective immunity and pathological outcomes in sepsis‐associated organ dysfunction.

#### 5.2.1 ILCs in Acute Lung Injury

**FIGURE 4:**
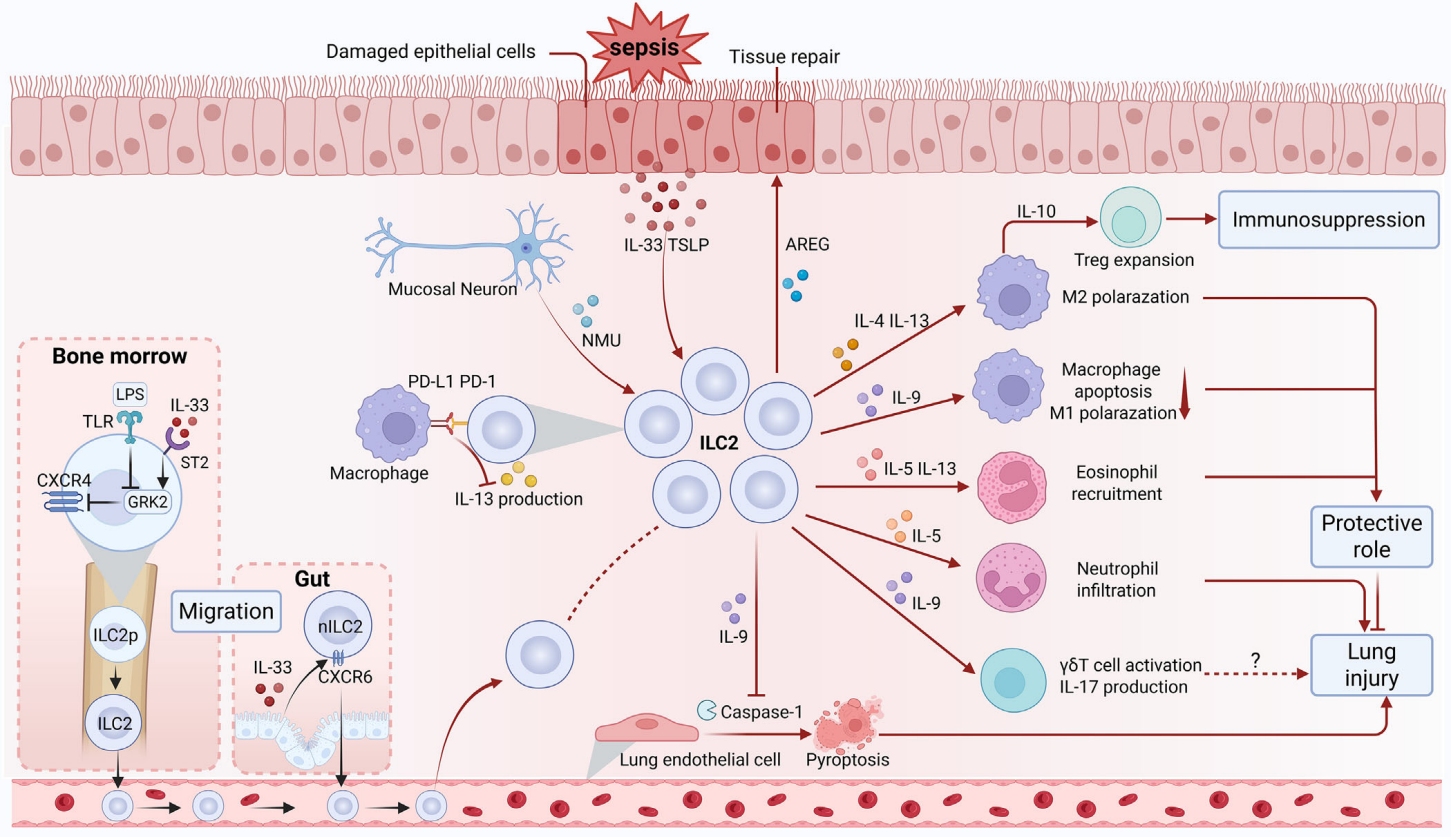
ILCs in sepsis‐associated acute lung injury. Damaged lung epithelial cells secrete IL‐33 and TSLP, while mucosal neurons release neuromedin U (NMU) to activate lung ILC2s. PD‐1 signaling negatively regulates ILC2s, particularly by suppressing IL‐13 production. IL‐33 acts on bone marrow ILC2 precursors (ILC2ps), increasing GRK2 expression, which in turn downregulates surface CXCR4 expression, thereby promoting ILC2 egress to the lungs (counteracted by LPS–TLR signaling). Gut‐derived natural ILC2s (nILC2s) migrate to the lung via IL‐33‐enhanced CXCR6‐mediated chemotaxis. Activated ILC2s secrete IL‐4 and IL‐13 to promote M2 macrophage polarization and IL‐10 production, driving Treg expansion and immunosuppression. ILC2‐derived IL‐5 and IL‐13 facilitate eosinophil recruitment, while IL‐9 suppresses macrophage apoptosis and M1 polarization and inhibits endothelial pyroptosis by suppressing caspase‐1 activation, thereby alleviating lung injury. IL‐9 also promotes γδ T cell activation and IL‐17A production (the effects on lung injury require investigation). IL‐5 exacerbates inflammation via neutrophil recruitment. ILC2‐derived amphiregulin (AREG) promotes epithelial repair (created with BioRender).

Sepsis‐associated acute lung injury (ALI), which is characterized by endothelial barrier disruption, alveolar epithelial damage, and microvascular thrombosis, is a common complication with high morbidity and mortality. Multiple mechanisms, such as the inflammatory cascade, oxidative stress, and coagulation dysfunction, contribute to the complex pathophysiology of ALI. Recent studies have demonstrated the activation and accumulation of ILC2s in the lungs during sepsis, which significantly affects ALI (Figure 4). As mentioned previously, pulmonary ILC2s constitute a highly heterogeneous population that primarily consists of ST2^+^IL‐17RB^+/−^KLRG1^+/−^ nILC2s, ST2^−^IL‐17RB^+^KLRG1^hi^ iILC2s, and IL‐10‐producing ILC2_10_s. Lai et al. revealed that sepsis induces increased expression of IL‐25, IL‐33, and TSLP as well as expansion of ILC2s in the lungs of mice [155]. However, only IL‐33 or ST2 deficiency blocked lung ILC2 expansion and reduced Type 2 cytokines, suggesting that these populations are predominantly nILC2s. Further studies have shown that sepsis leads to significant migration of ILC2 progenitor (ILC2p) from the BM into the lungs through IL‐33/ST2 signaling [156]. IL‐33 acts on ILC2ps in the BM to upregulate G protein‐coupled receptor kinase 2 (GRK2) expression. GRK2 downregulates surface CXCR4 (an important mediator that governs hematopoietic cell retention in the BM), thereby promoting ILC2 egress to the lungs [156]. Elevated ILC2s in the lungs produce IL‐9 to prevent lung endothelial cells from undergoing pyroptosis by inhibiting caspase‐1 activation, thereby attenuating the inflammatory response and lung injury [155]. ILC2‐derived IL‐9 can also prevent lung injury by suppressing macrophage apoptosis and M1 polarization through the inhibition of mTOR activation in sepsis models [135]. Intriguingly, Chen et al. revealed that IL‐9 promotes the expansion and subsequent production of IL‐17A by γδ T cells in septic lungs of mice; however, the impact of this axis on ALI still requires investigation [146]. Moreover, as previously mentioned, IL‐5 and IL‐13 can enhance eosinophil infiltration in the lungs, which is crucial for the ILC2‐mediated reduction in bacterial load and inflammation [43].

In contrast to the ILC2‐mediated protective effect, Xu et al. revealed that IL‐33‐elicited IL‐5 promotes the accumulation of neutrophils and monocytes in the lungs and exacerbates early inflammation‐induced ALI in abdominal sepsis models [138]. The absence of IL‐33 led to a greater intraperitoneal bacterial burden but less pulmonary damage [138]. Furthermore, in the later phase of sepsis, pulmonary ILC2‐derived IL‐4 and IL‐13 aggravate immunosuppression and increase the susceptibility to infectious pneumonia [45]. Therefore, the effect of ILC2s on the pathophysiology of ALI varies depending on the source of infection and the stage of sepsis. Notably, the PD‐1/PD‐L1 pathway inhibits ILC2 activation in septic lungs by suppressing STAT5 phosphorylation, especially the ability of ILC2s to produce IL‐13 [40]. IL‐33 activates ILC2s via ST2 and induces the expression of PD‐L1 in other cells, such as macrophages, resulting in negative feedback regulation, and their balance drives the dynamics of ILC2‐derived IL‐13 levels. Furthermore, Chen et al. demonstrated that sepsis increases NMU levels in the mice's lungs and promotes the expression of NMUR1 in ILC2s [146]. NMU is expressed primarily by mucosal neurons and induces ILC2 activation by regulating ILC2s downstream of a Ca2^+^/calcineurin/NFAT cascade and ERK1/2 phosphorylation [157]. Thus, targeting these regulatory molecules represents a promising strategy to maximize the benefits of ILC2s in ALI. Few studies have focused on ILC1s and ILC3s in sepsis‐associated ALI. Nevertheless, Muir et al. revealed that pulmonary ILC3s activated by IL‐23 are the dominant source of IL‐17 in the early stage of LPS‐induced ALI, and this cytokine exacerbates inflammatory damage by recruiting neutrophils [59]. Conversely, Felton et al. revealed that ILC3‐derived IL‐22 ameliorates LPS‐induced ALI by suppressing neutrophils and enhancing the epithelial barrier [142]. Additionally, endogenous PGE2 produced by lung cells, such as macrophages and damaged epithelial cells, can catalyze this cascade by activating ILC3s via the PGE2 receptor EP4 [142]. Furthermore, some studies have demonstrated the significant roles of ILC1s and ILC3s in clearing pulmonary infections, such as *Klebsiella pneumoniae*, influenza viruses, and *Pseudomonas aeruginosa*, via the secretion of IL‐17, IL‐22, or IFN‐γ, suggesting their potential role in preventing pneumonic sepsis [158, 159]. Thus, elucidating the pathogenic versus protective duality of ILC1s and ILC3s in septic ALI remains imperative.

#### 5.2.2 ILCs and Gut Barrier Defense

The gut is both a victim and amplifier of systemic inflammation. During sepsis, factors such as cytokine storms and IRI lead to disruption of the gut barrier and the dissemination of large amounts of PAMPs, which further exacerbate multiple organ dysfunction [160]. In the gut, ILCs critically maintain barrier integrity and microbial homeostasis; their dysfunction is a key factor driving the progression of sepsis. As mentioned previously, ILC3s regulate the Treg/Th17 balance and IgA production through cytokine secretion and MHC II‐dependent antigen presentation, thereby promoting tolerance to the commensal microbiota. Moreover, ILC3‐derived IL‐22 triggers mucin secretion by goblet cells and antimicrobial peptide secretion by Paneth cells while also regulating intestinal glycosylation to control dysbiosis [109]. ILC3s can integrate signals from microbial metabolites, including short‐chain fatty acids and retinoic acid, and bridge microbial ecology with host immune programming. During pathogen invasion, MNP‐derived IL‐23 and IL‐1β activate ILC3s, which strengthen intestinal barrier integrity, recruit neutrophils, and support MNP function by producing IL‐22, IL‐17, and GM‐CSF. ILC2s, which are activated by intrinsic enteric neuron‐derived NMU or IL‐33/IL‐25 from epithelia, secrete Type 2 cytokines and AREG to eliminate pathogens and promote tissue repair [38]. Additionally, ILCregs limit intestinal inflammatory damage by releasing IL‐10 to suppress inflammatory ILC1s and ILC3s [60].

**FIGURE 5:**
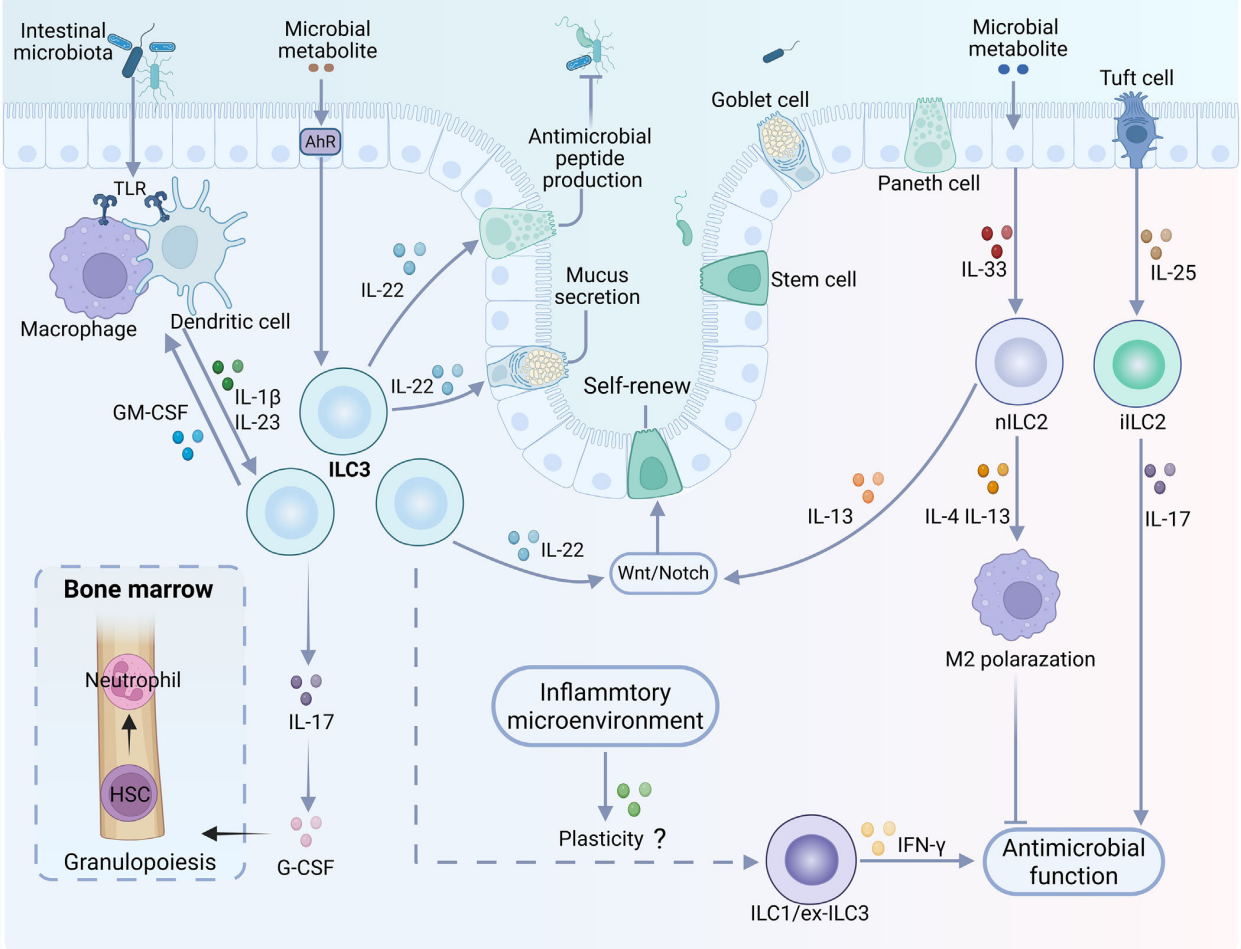
Intestinal ILCs regulate host resistance to sepsis. During infection, the gut microbiota can activate mononuclear phagocytes via TLR signaling, inducing the production of IL‐1β and IL‐23, which elicits ILC3‐derived IL‐17. IL‐17 recruits neutrophils and stimulates G‐CSF secretion to drive emergency granulopoiesis in the bone marrow, combating microbial infection. Additionally, microbial metabolites activate intestinal epithelial cells through the aryl hydrocarbon receptor (AHR), promoting NCR^+^ ILC3 expansion and IL‐22 release. IL‐22 enhances mucus secretion by goblet cells and antimicrobial peptide production by Paneth cells and also promotes intestinal stem cell self‐renewal via Wnt and Notch signals, facilitating barrier repair during sepsis. The inflammatory milieu in sepsis can drive ILC3 transdifferentiation into IFN‐γ‐secreting ILC1s through mechanisms that are not yet fully elucidated. Furthermore, gut microbiota‐induced epithelial IL‐33 stimulates ILC2 secretion of IL‐13, which synergizes with IL‐22 for tissue repair. Tuft cell‐derived IL‐25 activates inflammatory ILC2s (iILC2s) to produce IL‐17 for microbial defense, whereas natural ILC2s (nILC2s) promote M2 macrophage polarization via IL‐4/IL‐13, suppressing antimicrobial responses and increasing susceptibility to enterogenic sepsis (created with BioRender).

The role of ILCs in intestinal homeostasis and immune regulation has been well documented in other reviews [52]; here, we specifically highlight their capacity to modulate resistance to sepsis (Figure 5). Deshmukh et al. were the first to clarify the critical role of the gut microbiome‐ILC3 interplay in regulating resistance to neonatal sepsis [57]. The commensal microbiome‐activated TLR4/MYD88 signaling induces ILC3s to produce IL‐17A, which stimulates G‐CSF secretion to drive emergency granulopoiesis in the BM and promotes the release of mature neutrophils into the circulation [57]. This microbiome‐primed granulopoiesis establishes a frontline defense against bloodstream infections. Specific depletion of ILCs or disruption of microbes via antibiotic exposure impairs granulopoiesis and decreases resistance to sepsis, whereas transplantation of mature microbiota partially rescues neonatal mice from increased susceptibility [57]. Furthermore, emerging evidence suggests that milnacipran, an intestinal flora metabolite, promotes NCR^+^ ILC3 expansion and IL‐22 secretion by activating intestinal epithelial cells via the AhR signaling [58]. ILC3‐derived IL‐22 then enhances the self‐renewal of intestinal stem cells and the proliferation of epithelial cells through Wnt and Notch signals. This mitigates intestinal IRI‐induced damage, thereby alleviating the development of enterogenic sepsis in mice. Consistent with these findings, preoperative fecal milnacipran levels in cardiopulmonary bypass patients are negatively correlated with postoperative gastrointestinal injury risk [58]. Interestingly, another study reported a similar mechanism whereby the microbial metabolite pravastatin attenuates intestinal IRI by activating ILC2s [161]. Pravastatin promotes the production of IL‐33 by intestinal epithelial cells and induces ILC2s to secrete IL‐13. This cytokine enhances intestinal stem cell self‐renewal by activating Notch1 and Wnt signaling. [161]. In contrast to the protective effect mediated by ILCs, Ito et al. revealed that increased ILC2 numbers and Type 2 cytokine levels promote M2 macrophage polarization and suppress the host antibacterial function of macrophages, thereby facilitating sepsis stemming from enterococcal translocation [133]. Thus, the dysregulated activation of ILCs can paradoxically aggravate intestinal pathology during sepsis. These seemingly contradictory roles of intestinal ILCs underscore how the local tissue microenvironment, including the composition of the gut microbiota and the nature of the primary infection, can critically influence ILC function and ultimately determine sepsis outcome.

#### 5.2.3 Migration of ILCs via the Gut‒Lung Axis

Accumulating evidence indicates that the intestinal microbiota, metabolites, and immune cells can translocate to the lungs and exert pathogenic effects in ALI during sepsis. Interestingly, ILCs can also migrate from the gut into the lungs in response to specific stimuli. For example, the expression of the lung‐homing signaling molecule CCR4 on ILC3s induced by gut microbiota facilitates the lung‐selective trafficking of IL‐22^+^ ILC3s [162]. Disruption of commensal colonization impairs this migration and increases susceptibility of mice to pneumonia [162]. Furthermore, during LPS‐induced pulmonary inflammation, ligand‒receptor interactions between ILC3s and DCs mediated by CXCL16–CXCR6 facilitate ILC3 migration from the intestine to the lungs, thereby promoting resistance to pulmonary infections [163]. Indeed, recent studies suggest that lung iILC2s also originate from the intestines rather than being intrinsic to the lungs. After being stimulated by IL‐25 or intestinal infection, intestinal iILC2s enter the circulatory system, migrate to peripheral tissues, and accumulate mostly in the lung. These ILC2s may return to the intestine or transdifferentiate into lung‐resident nILC2s after the immune response subsides [164]. The migration of iILC2s relies on sphingosine 1‐phosphate‐mediated chemotaxis and is modulated by the basic leucine zipper ATF‐like TF [164, 165]. Notably, Pu et al. reported the ability of nILC2s to migrate through the gut‒lung axis in septic mice [166]. During sepsis, IL‐33 upregulates CXCR6 expression in nILC2s and promotes their migration from the gut to the lung via the CXCR6‒CXCL16 chemotaxis axis, whereas IL‐25 induces iILC2 accumulation in the intestine through the CCR9‒CCL25 axis [166]. These findings partially explain the scarcity of detectable ILC2 expansion in septic lungs. Pharmacologic inhibition of CXCR6 and CCR9 disrupts ILC2 circulation and exacerbates pulmonary and intestinal damage, indicating the necessity of ILC2 gut–lung migration for host defense in sepsis.

#### 5.2.4 ILCs in Cardiac Dysfunction

**FIGURE 6:**
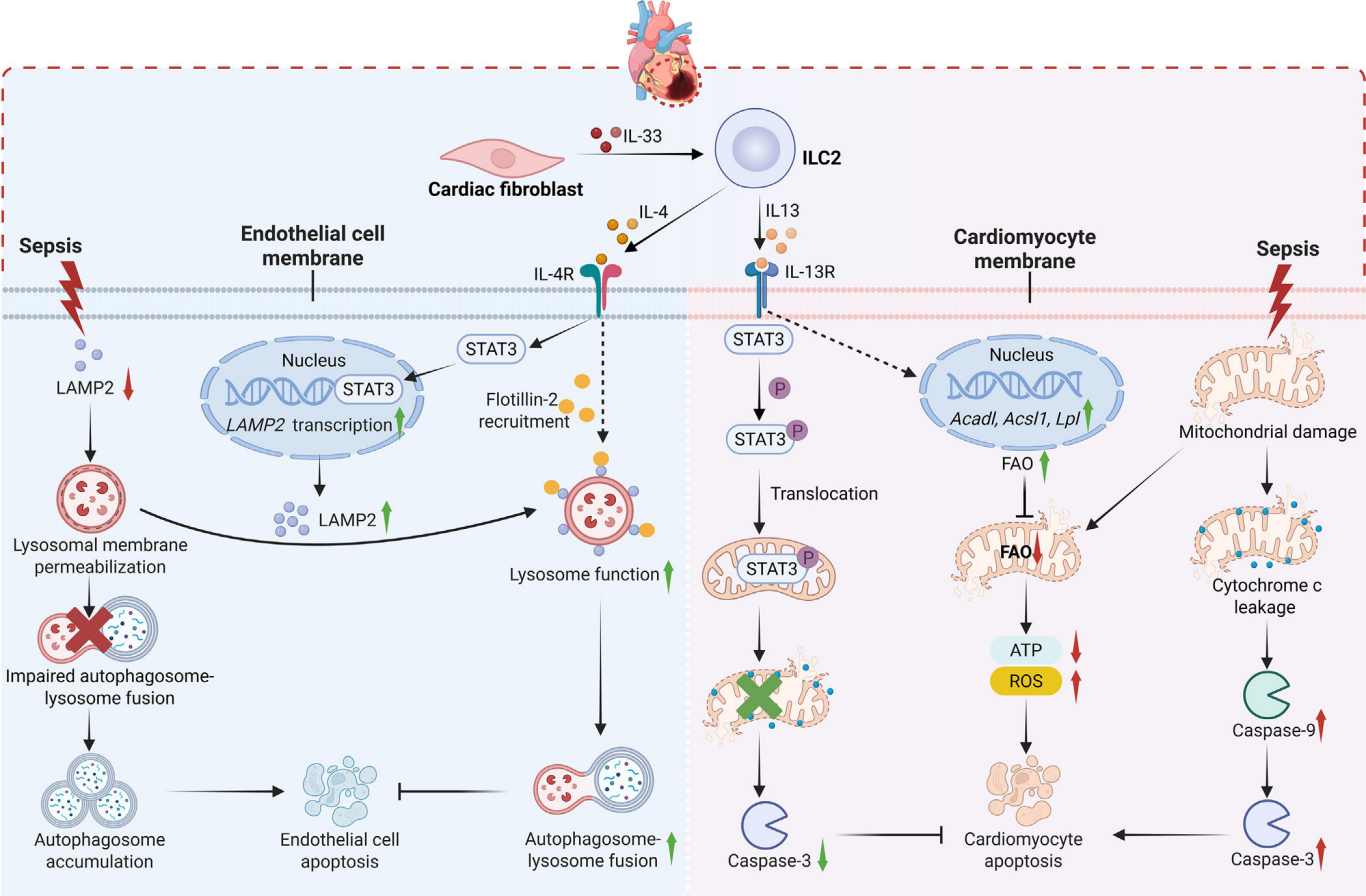
Protective role of ILC2s in sepsis‐induced cardiac dysfunction. Sepsis triggers IL‐33 release from cardiac fibroblasts, activating resident ILC2s to secrete IL‐4 and IL‐13. ILC2‐derived IL‐4 activates STAT3 in cardiac endothelial cells, leading to the upregulation of LAMP2 transcription. LAMP2–flotillin‐2 interaction prevents lysosomal membrane permeabilization and rescues impaired autophagic flux by facilitating autophagosome–lysosome fusion, thereby attenuating endothelial apoptosis. IL‐13 enhances STAT3 phosphorylation and promotes its mitochondrial translocation, suppressing cytochrome c leakage and caspase‐3‐mediated cardiomyocyte apoptosis. IL‐13 also upregulates fatty acid oxidation (FAO) genes (e.g., *Acadm*, *Acsl1*), enhancing mitochondrial energy metabolism and reducing reactive oxygen species (ROS) production in cardiomyocytes, thereby preserving cardiac function (created with BioRender).

ILC2s constitute the predominant subgroup of ILCs in cardiac tissue, while the proportions of ILC1s and ILC3s are significantly lower [167]. Under steady‐state conditions, cardiac ILC2s exhibit quiescent progenitor‐like features, such as intermediate GATA‐3 expression levels and the capacity to transiently express PLZF upon activation [168]. They differentiate into conventional ILC2s in response to inflammatory or ischemic stimuli via IL‐33 signaling from cardiac fibroblasts and exhibit unique phenotypes, such as low expression of ICOS/CD25 and heightened IL‐4/IL‐13 production capacity [167, 168]. Cardiac ILC2s lack IL‐25 receptor (IL‐17RB) expression and cannot transdifferentiate into iILC2s. Recent studies have revealed that ILC2s critically protect against sepsis‐induced cardiac injury, which is a life‐threatening complication driven by complex mechanisms such as a dysregulated inflammatory response, mitochondrial dysfunction, and excessive nitric oxide production [169, 170]. During sepsis, the oxidative damage caused by DAMPs to mitochondrial DNA, the overexpression of NOS leading to increased permeability, incomplete autophagy, and other mechanisms result in mitochondrial dysfunction, contributing to reduced energy production and cardiomyocyte “hibernation” [169]. Recent findings highlight the role of ILC2‐derived IL‐13 in reversing sepsis‐induced mitochondrial disorders to mitigate heart injury. In LPS‐induced sepsis models, ILC2s are identified as the primary source of IL‐13 in cardiac tissues [171]. IL‐13 enhances STAT3 Ser727 phosphorylation through Jak signaling downstream of IL‐13Rα1 and promotes its transfer into the mitochondria, thereby suppressing cytochrome c leakage and caspase‐3‐mediated cardiomyocyte apoptosis induced by sepsis [126]. Moreover, sepsis‐induced mitochondrial fatty acid oxidative damage impairs cardiac function, whereas IL‐13 upregulates genes (e.g., *Acadl*, *Acsl1*) that are critical for fatty acid uptake and β‐oxidation, enhancing mitochondrial energy metabolism in cardiomyocytes [127]. IL‐13 supplementation alleviates damage to the electronic delivery chain induced by LPS, reduces ROS overproduction, and stabilizes the mitochondrial membrane potential, whereas ILC2 depletion abrogates IL‐13 production, exacerbating cardiomyocyte apoptosis and cardiac dysfunction [172]. In addition to direct mitochondrial modulation, emerging evidence demonstrates that ILC2s orchestrate cardiac protection by regulating autophagy, which is crucial for clearing damaged organelles and preventing the release of apoptotic signaling factors from compromised structures. In the early stage of sepsis, cardiac ILC2s significantly expand and produce high levels of IL‐4, which activates STAT3 to upregulate lysosomal‐associated membrane protein 2 (LAMP2) in cardiac endothelial cells [173]. Elevated LAMP2 stabilizes lysosomal integrity, prevents lysosomal membrane permeabilization, and promotes autophagosome–lysosome fusion through direct interaction with the lipid raft protein flotillin‐2, thereby mitigating sepsis‐induced inflammation and apoptosis caused by autophagic flux blockade. Notably, IL‐4 also attenuates mitochondrial damage [173]. This observation suggests that IL‐4 may synergistically maintain mitochondrial function with IL‐13 by promoting mitochondrial autophagy as an indirect consequence of enhanced general autophagic flux. Moreover, Fang et al. revealed that the number of IL‐5^+^ ILC2s increases in the heart of mice during sepsis and that IL‐5 protects cardiomyocytes from apoptosis induced by incomplete autophagic flux; however, the underlying mechanism remains to be further elucidated [174]. In addition, cardiac fibroblast‐derived IL‐33 is essential for ILC2 expansion and activation, and its depletion abolishes ILC2‐mediated protection against sepsis‐induced heart injury [168, 174] (Figure 6).

ILC2s may also protect against cardiac injury through interactions with other immune cells. In myocardial infarction and myocarditis, ILC2 was shown to regulate downstream immune responses via cytokines, including IL‐4, IL‐5, and IL‐13 [116, 175, 176, 177]. These cytokines orchestrate eosinophil recruitment, DC‐mediated Treg activation, and macrophage M2 polarization, collectively suppressing inflammation, reducing cardiomyocyte death, and enhancing tissue repair to improve cardiac function. In an LPS‐induced sepsis mouse model, IL‐5 deficiency exacerbates cardiac injury by promoting M1 macrophage differentiation through activation of the NF‐κB–p65 pathway [134]. Finally, although ILC2s in the heart are primarily tissue‐resident and rarely replenished by circulating cells under physiological conditions, it is unclear whether ILC2s from other organs migrate to damaged heart tissue under pathological conditions such as sepsis.

#### 5.2.5 ILCs in Other Tissues

The extensive distribution of ILCs throughout the body suggests their potential roles in various tissues, not merely in the aforementioned organs. For example, ILC2‐derived IL‐13 in skeletal muscle reverses the sepsis‐induced downregulation of slow‐twitch fiber‐specific genes, restoring the transcription of myoglobin and troponin I Type 1 to counteract atrophy and weakness during sepsis [178]. This mechanism, while mechanistically defined in preclinical models, highlights a potential pathway relevant to sepsis‐associated myopathy; its clinical correlation, such as with specific immune markers in patients with muscle weakness, remains to be established. Moreover, several studies have documented alterations in ILCs within the liver during sepsis. In the early stages of sepsis (24 h post‐CLP), there is a significant reduction in hepatic ILC2 and ILC1 numbers, which is accompanied by increased mitochondrial activity and capacity to secrete IFN‐γ in ILC1s [44, 179]. In addition, as previously discussed, the mechanism by which the ILC1–imNeu axis promotes imNeu migration and IL‐10 production via the IFN‐γ–CXCR4 signaling pathway has been demonstrated to ameliorate liver injury in a mouse model of hepatic IRI, suggesting a potential conserved pathway for limiting inflammation‐induced organ damage [140]. However, translating these findings to the clinical context of sepsis requires caution. Further investigation of the corresponding clinical observations linking specific hepatic ILC changes to sepsis outcomes in patients is warranted. ILC1‐derived IFN‐γ has been demonstrated to negatively modulate endotoxin tolerance in sepsis [180]. Interestingly, the role of ILC1‐derived IFN‐γ in sepsis appears to be complex and context dependent. For instance, in LPS‐induced sepsis mouse models, liver and spleen group 1 ILCs expressed glucocorticoid receptors (GRs), while depletion of GRs augmented IFN‐γ production by ILC1s and subsequently decreased the serum levels of IL‐10, thereby increasing the susceptibility to septic shock [180]. These findings suggest that IFN‐γ inhibits the secretion of IL‐10 by myeloid cells, whereas endogenous glucocorticoids suppress ILC1‐derived IFN‐γ via GR signaling, thus maintaining shock resistance. This highlights a critical immunoregulatory checkpoint where glucocorticoids fine‐tune the ILC1 response. Notably, the relative contribution of NK cells versus ILC1s in this pathway remains unresolved and warrants further investigation.

In summary, ILCs function as central orchestrators of sepsis pathophysiology, integrating immune responses across tissues through cytokine production and cellular crosstalk. Their dual roles underscore that ILC functions are not merely protective or pathogenic but are dynamically shaped by the tissue microenvironment, phase of sepsis, and host‐specific factors. A major future challenge lies in deciphering how patient heterogeneity dictates ILC responses. Future research must move beyond characterizing ILCs in “average” sepsis models and embrace stratified approaches. Longitudinal studies tracking ILC dynamics in patients stratified by infection type, age, and genetic markers are urgently needed. Furthermore, developing animal models that incorporate relevant human comorbidities (e.g., diabetes and aging) is essential for understanding how the presepsis immune landscape influences ILC function. Ultimately, personalized ILC‐targeted therapies depend on our ability to predict an individual's ILC response profile based on these multifaceted variables.

## 6 ILCs beyond Sepsis: Lessons from Other Diseases

**FIGURE 7:**
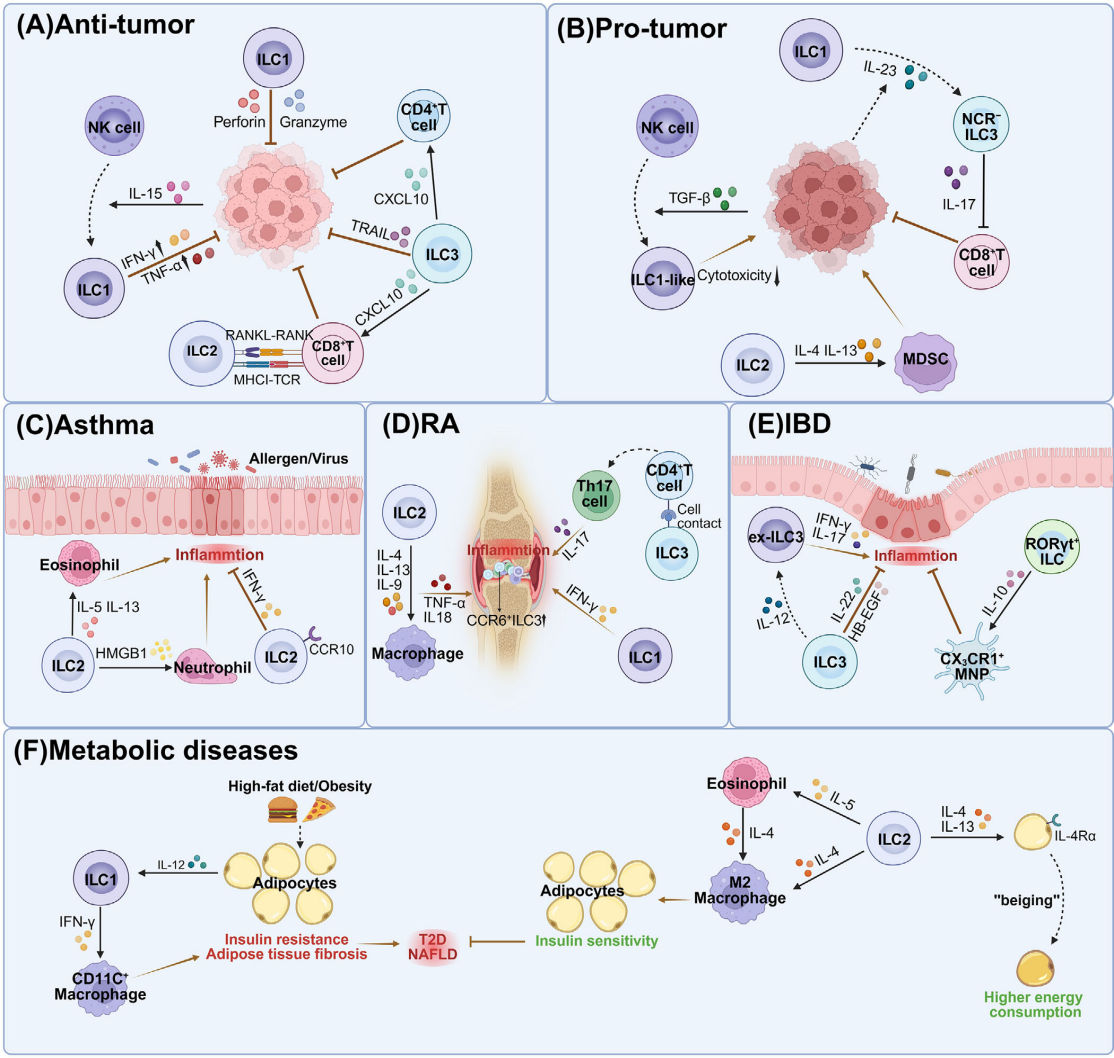
Role of ILCs in various diseases. (A) Protumor functions of ILCs. Cancer‐derived IL‐15 induces the differentiation of IFN‐γ/TNF‐α‐rich ILC1‐like cells with enhanced antitumor activity. ILC1s can also exert direct cytotoxicity via granzyme/perforin. ILC2s prime CD8^+^ T cells via MHC‐I and RANK–RANKL signaling. ILC3s recruit T cells via CXCL10 and induce tumor cell apoptosis via TRAIL. (B) Antitumor functions of ILCs. TGF‐β converts NK cells into hypofunctional ILC1s. ILC2s recruit myeloid‐derived suppressor cells (MDSCs) via IL‐4/IL‐13. IL‐23 drives the differentiation of IL‐17‐producing ILC3s that suppress CD8^+^ T cell activity. (C) ILCs in asthma. ILC2s drive pathology by releasing IL‐5/IL‐13 to recruit eosinophils and by releasing HMGB1‐loaded extracellular droplets to recruit neutrophils. A protective CCR10^+^ ILC2 subset secreting IFN‐γ has also been identified. (D) ILCs in rheumatoid arthritis (RA). ILC2s exert anti‐inflammatory effects via IL‐4/IL‐9/IL‐13 to modulate macrophages. Conversely, ILC3s promote inflammation by interacting with T cells to drive Th17 differentiation. CCR6^+^ ILC3s accumulate in inflamed joints. ILC1s exacerbate inflammation via IFN‐γ. (E) ILCs in inflammatory bowel disease (IBD). IL‐12 drives the generation of inflammatory ex‐ILC3s secreting IFN‐γ/IL‐17. Protective ILC3 subsets secrete IL‐22 and HB‐EGF for tissue repair, and RORγt^+^ ILCs can produce anti‐inflammatory IL‐10. (F) ILCs in metabolic diseases. ILC1s, activated by adipocyte‐derived IL‐12, secrete IFN‐γ to polarize M1 macrophages, thereby promoting insulin resistance, tissue fibrosis, and exacerbating metabolic diseases such as Type 2 diabetes (T2D) and nonalcoholic fatty liver disease (NAFLD). ILC2s improve metabolic health by driving white adipose tissue beiging via IL‐4/IL‐13 and by promoting M2 macrophages via IL‐4/IL‐5 to enhance insulin sensitivity (created with BioRender).

The intricate roles of ILCs in sepsis, as detailed in the preceding chapter, provide a compelling paradigm of how these cells integrate immune responses in dynamic and life‐threatening conditions. However, the immunological principles governing ILC function extend far beyond the context of sepsis. Examining ILCs in a broader spectrum of pathological conditions reveals conserved mechanisms of immune regulation and highlights the remarkable plasticity and context dependency of ILC responses across different tissue microenvironments. This section provides an review of the role of ILCs in different diseases, including cancer, inflammatory diseases, and metabolic disorders (Figure 7).

### 6.1 Cancer

The immune system engages in a dynamic struggle with developing tumors, a process historically framed as cancer immunosurveillance. However, it is now clear that the tumor microenvironment (TME) can subvert protective immunity, leading to immune evasion and even exploitation of immune cells for protumorigenic purposes. ILCs, as key tissue‐resident immune sentinels, exemplify this duality, with their function being critically shaped by the TME [181, 182]. Unlike conventional NK cells that directly eliminate target cells through perforin or Gzm release and antibody‐dependent cellular cytotoxicity, ILC1s primarily influence antitumor immunity through the secretion of cytokines such as IFN‐γ [183]. Genetic models selectively depleting liver‐resident ILC1s or conventional NK cells reveal a functional division: ILC1s are critical for inhibiting hepatic metastatic seeding, whereas NK cells primarily restrict tumor outgrowth [184]. This functional specialization underscores a nonredundant collaboration between Group 1 ILCs within the TME. In head and neck squamous cell carcinoma, cancer cell‐derived IL‐15 drives the differentiation of circulating NK cells into a CD49a^+^CD103^+^ intraepithelial ILC1‐like subset characterized by high expression of IFN‐γ and TNF‐α, which confers a heightened capacity to control tumor growth [185]. Furthermore, accumulating evidence has demonstrated that ILC1s can exert antitumor effects via cytotoxic mechanisms. In chromophobe renal cell carcinoma, the presence of tumor‐infiltrating, Gzm A‐expressing ILC1s is significantly associated with longer patient survival, highlighting their clinical relevance [186]. Complementing these human data, research in mouse models of breast cancer has shown that ILC1s expand and express Gzm C upon IL‐15 stimulation, equipping them with perforin‐dependent cytotoxic activity capable of mediating effective cancer immunosurveillance [187]. In addition to IL‐15 signaling, NKp46 serves as a critical regulator of ILC1s [188]. Antibody‐mediated activation of NKp46 enhances ILC1 effector functions, whereas its deletion abrogates the control of tumor growth in acute myeloid leukemia models [188]. However, several findings indicate that ILC1s can become functionally impaired and exhibit protumor activity in certain tumors. In a murine fibrosarcoma model, TGF‐β signaling drives the conversion of tumor‐suppressive NK cells into intermediate ILC1s (CD49a^+^CD49b^+^Eomes^+^) and noncytotoxic ILC1s [189]. These ILC1s secrete less IFN‐γ and produce elevated levels of TNF, which collectively diminishes antitumor immunosurveillance and accelerates tumor growth [189]. In melanoma patients, elevated proportions of circulating ILC1s are associated with resistance to immunotherapy, suggesting the potential protumor activity of ILC1s in human cancers [190]. These findings challenge the traditional view that ILC1s primarily exert antitumor functions.

ILC2s play context‐dependent roles in cancer, exhibiting both protumorigenic and antitumorigenic functions across different tumor types and microenvironments. On the one hand, ILC2s can promote immunosuppression and tumor progression. For instance, in breast cancer models, pre‐existing Type‐2 airway inflammation promotes lung metastatic seeding via an IL‐33/ILC2‐dependent pathway [191]. Mechanistically, ILC2s suppress innate antitumor immunity by recruiting and activating eosinophils in an IL‐5‐dependent manner; these eosinophils directly inhibit NK cell function by altering the metabolic niche, thereby enabling immune evasion [191]. Similarly, in colorectal cancer (CRC), IL‐25‐activated ILC2s foster a tumor‐permissive microenvironment by producing IL‐4 and IL‐13, which recruit and sustain myeloid‐derived suppressor cells (MDSCs), thereby suppressing antitumor immunity [192]. Consistent with the results of animal experiments, high levels of IL‐25 in CRC tumors show an adverse association with patient survival [192]. Research also reveals that a feed‐forward signaling circuit between gastric tuft cells and ILC2s, mediated by IL‐25 and IL‐13, is co‐opted from its physiological role in epithelial repair to drive metaplasia and tumorigenesis in the stomach [193]. Clinically, signatures of this tuft cell–ILC2 axis are associated with poorer survival in patients with intestinal‐type gastric cancer [193]. Furthermore, in prostate cancer, patients exhibit a stage‐dependent increase in protumoral ILC2s and a concomitant reduction in antitumoral ILC1s, with ILC2 frequency correlating with higher prostate‐specific antigen levels, suggesting its prognostic value [194]. On the other hand, ILC2s have also been widely reported to exert antitumor effects. In a melanoma model, IL‐33 exerts an antitumor effect by engaging ILC2s, which in turn activate CD8^+^ T cells via the OX40L–OX40 costimulatory pathway [195]. ILC2s have also been identified to exert antitumorigenic function in melanoma by recruiting and activating antitumor eosinophils via GM‐CSF [196]. Intriguingly, a recent study revealed that ILC2s can directly prime CD8^+^ T cells through MHC‐I‐restricted antigen presentation and RANK–RANKL costimulatory signaling, leading to cytotoxic T cell differentiation and enhanced tumor‐killing activity [197]. Clinically, high infiltration of ILC2s is associated with a favorable patient prognosis in multiple tumor types, such as pancreatic ductal adenocarcinoma, hepatocellular carcinoma (HCC), and melanoma [196, 198, 199, 200]. Moreover, human ILC2s exhibit a previously unappreciated direct cytotoxic function against tumors by secreting Gzm B, a process activated through the DNAM‐1 signaling pathway [201]. These ex vivo expanded cytotoxic ILC2s present a promising avenue for cell‐based immunotherapy [201]. Overall, the functional plasticity of ILC2s underscores their potential as immunotherapeutic targets, although their precise modulation requires a nuanced understanding of context‐dependent signals and mechanisms.

The role of ILC3s in CRC has garnered significant interest, yet their functions remain complex and context dependent. IL‐22 production by ILC3s can promote epithelial repair and protect stem cells during early tumorigenesis, whereas ILC3 dysregulation and plasticity toward protumorigenic phenotypes can contribute to chronic inflammation and tumor growth [52]. ILC3s are reduced in human and murine tumors in CRC compared with normal tissue and exhibit plasticity through the acquisition of ILC1‐associated markers [202]. Functionally, MHCII^+^ ILC3 depletion accelerates tumor progression and impairs the response to anti‐PD‐1 therapy, suggesting their protective role in antitumor immunity [202]. In murine cold tumor models, cisplatin‐induced production of CCL20 and IL‐1β recruits and activates CCR6^+^ ILC3s, which in turn secrete CXCL10 to drive the infiltration of CD4^+^ and CD8^+^ T cells [203]. This ILC3‐dependent T cell recruitment is essential for antitumor immunity and enhances the efficacy of checkpoint inhibition [203]. Beyond their role in immune cell recruitment, ILC3s can directly exert antitumor cytotoxicity against various carcinomas, including HCC and melanoma, through IFN‐γ production and TRAIL‐mediated activation of Caspase‐8 in target cells [204]. Conversely, under specific microenvironmental conditions, ILC3s can undergo phenotypic conversion that promotes tumor progression. In HCC, elevated IL‐23 expression expands NCR^−^ ILC3s and drives their differentiation from ILC1s, leading to the production of IL‐17, which directly suppresses CD8^+^ T cell immunity via the induction of apoptosis and proliferation arrest, thereby fostering an immunosuppressive TME and accelerating tumor progression [205]. Furthermore, gut dysbiosis promotes the protumor function of ILC3s by epigenetically enhancing their production of IL‐17A via decreased SOX13 acetylation [206]. This ILC3‐driven inflammatory response contributes to HCC progression and is correlated with poorer patient outcomes [206]. The protumorigenic capacity of ILC3s is also evident in familial adenomatous polyposis, where duodenal adenomas exhibit accumulation of IL‐17A^+^NKp44^−^ ILC3s driven by elevated IL‐1β, IL‐23A, and DLL4 signaling [207]. These ILC3s promote a tumorigenic microenvironment by stimulating DUOX2/DUOXA2‐dependent ROS production and DNA damage in epithelial cells, thereby increasing susceptibility to oncogenic transformation [207]. In summary, ILC3s act as a double‐edged sword in tumor immunity, with their functional outcomes critically dependent on microenvironmental signals, subset specificity, and crosstalk with other immune cells. Unraveling their regulatory mechanisms holds promise for developing precision immunotherapeutic strategies.

### 6.2 Inflammatory Diseases

Inflammatory diseases, characterized by dysregulated and often persistent immune activation, represent a major area in which ILCs exert critical influence. In conditions such as asthma, rheumatoid arthritis (RA), and inflammatory bowel disease (IBD), the line between protective immunity and pathological inflammation is frequently disrupted. ILCs are central players in this balance, and their functions shift from maintaining tissue homeostasis in health to driving or modulating chronic inflammation in disease. This section delves into the complex and often dualistic roles of ILC subsets in major inflammatory diseases, highlighting how their contributions can be both pathogenic and protective.

#### 6.2.1 Asthma

Asthma is a prevalent chronic respiratory disorder characterized by pathological features such as airway hyperreactivity (AHR), persistent inflammation, and structural remodeling of the airways. This heterogeneous disease is typically classified into Type 2‐high (T2‐high) and Type 2‐low (T2‐low) endotypes [208]. The T2‐high immune response is mediated primarily by Type 2 cytokines and their effector cells, particularly Th2 cells and ILC2s [209]. Upon exposure to common allergens and proteases, such as *Alternaria alternata*, house dust mites, and papain, ILC2s are activated and release substantial quantities of IL‐13 and IL‐5 [208, 210]. These cytokines drive key asthma‐like pathological features, including eosinophil infiltration, AHR, and mucus hypersecretion. Beyond their well‐established role in eosinophilic asthma, recent evidence indicates that ILC2s also contribute critically to T2‐low asthma by promoting neutrophil infiltration, a hallmark of severe disease. A recent study demonstrated that IL‐33‐activated ILC2s release extracellular lipid droplets loaded with the alarmin HMGB1, which in turn triggers neutrophil recruitment via the CXCL2–CXCR2 axis, thereby exacerbating airway inflammation [139]. Clinical studies further support the relevance of ILC2s in asthma. Increased ILC2 frequencies have been observed in the sputum, peripheral blood, and bronchoalveolar lavage fluid of patients with asthma [211]. Consistently, elevated levels of ILC2‐activating cytokines—IL‐33, TSLP, and IL‐25—have been detected in the bronchial epithelium of these patients [212, 213]. Moreover, ILC2 numbers and Type 2 cytokine levels are positively correlated with disease severity [211, 214, 215]. In murine models of acute asthma, ILC2s exhibit memory‐like properties and have been identified as key contributors to disease relapse, serving as a major source of Type 2 cytokines with significantly enhanced production during recurrent episodes [216]. Mechanistically, the recurrence of acute allergic asthma is driven, at least in part, by ILC2s in an IL‐33/IL1RL1‐dependent manner [216]. Additionally, glucocorticoid resistance in ILC2s represents an important mechanism underlying refractory asthma. Studies have shown that lung ILC2s remain activated and sustain chronic inflammation even after corticosteroid treatment [217]. Emerging therapeutic agents, such as JAK3 inhibitors, have been shown to ameliorate steroid‐resistant asthma by suppressing ILC2 proliferation and cytokine production [218]. Notably, a recent study revealed that CCR10^+^ ILC2s are elevated in the circulation of patients with severe asthma and are recruited to lung tissues, concomitant with increased plasma levels of CCL27 [219]. In mouse models, these CCR10^+^ ILC2s acquire ILC1‐like properties by secreting IFN‐γ, which exerts immunomodulatory effects and confers protection against allergic airway inflammation [219].In summary, the role of ILC2s in asthma remains controversial. A deeper understanding of ILC2 heterogeneity is imperative for paving the way for effective targeted therapies against asthma.

#### 6.2.2 Rheumatic Arthritis

RA is a chronic autoimmune disorder characterized by persistent synovial inflammation of multiple joints. A growing body of evidence has revealed significant alterations in ILC subset distributions in this disease, suggesting their potential involvement in immunomodulation. Analysis of patients with stable RA reveals a distinct peripheral blood ILC profile, characterized by elevated ILC2 frequencies alongside reduced ILC1 and ILC3 populations [220]. Importantly, the ILC1 proportion positively correlated with disease activity, whereas ILC2 frequency shows a negative correlation. These findings, corroborated in a murine arthritis model, indicate that ILC2s may have a protective effect on RA [220]. ILC2s are considered to exert their anti‐inflammatory functions primarily via IL‐4, IL‐9, and IL‐13, which act by modulating macrophages, thereby ameliorating synovitis in models of RA [221, 222]. Conversely, ILC3s appear to play a proinflammatory role. In both collagen‐induced arthritis mouse models and RA patients, CCR6^+^ ILC3s are enriched within inflamed joints and exhibit elevated expression of proinflammatory cytokines [223]. Their levels show a positive correlation with clinical joint scores and the concentration of their ligand, CCL20 [223]. Furthermore, compared with healthy controls, patients with RA exhibit elevated frequencies of Th17 cells and ILC3s, accompanied by increased serum levels of IL‐17A and IL‐22 [224]. This proinflammatory axis is further supported by work from Liu et al., showing that direct interactions between ILC3s and T cells promote Th17 differentiation in RA [225]. Collectively, these findings suggest that ILC3s likely promote pathogenic inflammation in RA. The role of ILC1s in RA, however, remains an area of ongoing debate and appears context dependent. For instance, Lo Pizzo et al. reported that RA patients exhibit increased peripheral blood ILC1s and demonstrated that tofacitinib (a JAK inhibitor) can modulate this innate immune response by suppressing ILC1 frequency and IFN‐γ production [226]. In contrast, recent work by Arra et al. demonstrated that in patients with active RA, the frequency of ILC3s is elevated while that of ILC1s is reduced—a phenomenon potentially linked to the conversion of ILC1s to ILC3s [227]. Notably, treatment with the JAK inhibitor baricitinib appears to restore ILC homeostasis, and the therapeutic response is correlated with changes in PD‐1^+^ ILC3 frequency [227]. These discrepant findings may arise from differences in patient cohorts, disease activity (e.g., stable vs. active RA), or detection methodologies.

#### 6.2.3 Inflammatory Bowel Disease

IBD, encompassing Crohn's disease and ulcerative colitis, represents a group of chronic and relapsing inflammatory conditions of the gastrointestinal tract. It poses a significant and growing global health burden, with increasing incidence worldwide, particularly in newly industrialized regions. Numerous studies have reported the multifaceted roles of ILC3s in IBD, ranging from barrier protection to promotion of chronic inflammation. In patients with Crohn's disease and ulcerative colitis, the frequency of intestinal ILC3s—particularly the NKp44^+^ subset—is significantly reduced, and this reduction is correlated with disease severity [228, 229, 230]. Functional impairment of ILC3s has also been observed, characterized by decreased production of protective factors such as IL‐22, IL‐2, and HB‐EGF, thereby exacerbating intestinal inflammation [53, 101, 231, 232]. Moreover, ILC3s exert beneficial effects on IBD by expressing surface molecules such as MHC II and cytotoxic T‐lymphocyte‐associated antigen‐4 (CTLA‐4), through which they support Tregs and restrain inflammatory T cell responses [98, 233]. However, some studies suggest a context‐dependent role of ILC3s: for instance, IL‐22 aggravates colonic pathology by promoting endoplasmic reticulum stress in epithelial cells [234]. Conversely, inhibition of GM‐CSF production in colonic ILC3s was found to ameliorate colitis [235]. These findings collectively suggest a dual role of ILC3s in IBD pathogenesis. Notably, an increased ILC1/ILC3 ratio has been observed in IBD, implying possible conversion of ILC3s to ILC1s or inflammatory ex‐ILC3s that secrete IFN‐γ and IL‐17, thereby amplifying intestinal inflammation [52, 228, 236]. Furthermore, a recent study using an anti‐CD40 colitis model identified a subset of RORγt^+^ ILC3s that alleviates inflammation by producing IL‐10 to modulate intestinal macrophages—though it remains unclear whether this subset exists in human IBD patients [237]. In summary, substantial changes in ILC3 number and function occur in IBD, suggesting that restoring ILC3 populations could help restrain pathogenic T cells, promote barrier repair, and induce mucosal healing. Discrepancies in methodology—such as tissue selection, cellular heterogeneity, and gating strategies—may explain the conflicting reports on ILC3 dynamics in human IBD. Future studies using standardized approaches and well‐defined cohorts are needed to clarify the role of ILC3 alterations in this disease.

### 6.3 Metabolic Diseases

The increasing global burden of metabolic diseases, including obesity, T2D, and nonalcoholic fatty liver disease (NAFLD), underscores the critical need to elucidate the complex pathogenic mechanisms beyond traditional paradigms. The field of immunometabolism has revolutionized our understanding of these metabolic disorders, revealing a central role for ILCs.

Research has demonstrated that the population of ILC1s expands within the adipose tissue of both HFD‐fed mice and human patients with T2D [238, 239, 240]. The accumulation of these adipose tissue‐resident ILC1s contributes to local inflammation and systemic insulin resistance. Mechanistically, HFD consumption prompts adipocytes to produce IL‐12, which in turn drives the proliferation of adipose ILC1s and their production of IFN‐γ in a STAT4‐dependent manner. This ILC1‐derived IFN‐γ subsequently promotes the polarization of proinflammatory M1 macrophages and suppresses the IL‐33‐triggered ILC2 response, thereby exacerbating obesity‐associated insulin resistance [241]. Furthermore, activated ILC1s stimulate M1 macrophages to release TGF‐β1, which promotes adipose tissue fibrosis [238]. Importantly, the neutralization of IL‐12 to block ILC1 accumulation has been shown to ameliorate adipose tissue fibrosis, improve glucose tolerance, and reduce hepatic steatosis in HFD‐fed mice [238]. Additionally, a recent study revealed that ILC1 deficiency improves glucose metabolism and insulin sensitivity through multiple mechanisms, including enhanced activation of ILC3s and the IL‐22 pathway, which improves gut barrier integrity, and increasing the number of intestinal endocrine cells that modulate insulin secretion [242]. However, some studies suggest that ILC1s may also play beneficial roles in metabolic homeostasis. For instance, a study reported that high levels of TGF‐β in the livers of obese mice can drive a phenotypic and functional shift of NK cells into less cytotoxic ILC1‐like cells [243]. This conversion confers protection against NAFLD [243]. Furthermore, work by Boulenouar et al. indicated that under steady‐state conditions, adipose‐resident ILC1s help maintain tissue homeostasis by eliminating macrophages via cytotoxic activity [244]. In diet‐induced obesity, both the number and cytotoxic function of ILC1s are impaired, leading to pathogenic macrophage accumulation and exacerbated metabolic dysfunction [244]. These findings collectively highlight a context‐dependent duality in ILC1 functions, suggesting that their role in metabolic health is complex and may vary based on the tissue microenvironment and metabolic status.

As previously discussed, ILC2s play a beneficial role in regulating metabolic homeostasis, and their activation has been demonstrated to be closely associated with the amelioration of various metabolic diseases. A study showed that ILC2s alleviate metabolic disorders induced by an high‐fat high‐sucrose diet through the CD36‐dependent limitation of saturated fatty acid accumulation in adipose tissue [245]. Deficiency of ST2 decreased ILC2s in WAT, whereas ex‐ILC2s, which acquired ILC1‐like traits, increased [245]. This led to significant metabolic disorders such as visceral fat obesity, reduction of energy metabolism, and impaired glucose tolerance. Death receptor 3 (DR3), a member of the TNF superfamily, has been identified as a key regulator of ILC2s that is upregulated by IL‐33 [246]. DR3 signaling through NF‐κB pathways enhances the function of ILC2s in adipose tissue and peripheral blood, leading to improved glucose metabolism and insulin resistance [246]. Engagement of GITR, another member of the TNF superfamily, on ILC2s has also been shown to promote Type 2 cytokines, thereby ameliorating insulin resistance and preventing T2D [247]. The cannabinoid receptor 2 (CB2), a G‐protein‐coupled receptor, is expressed on ILC2s from both murine and human visceral adipose tissue [248]. CB2 engagement on ILC2s enhances Type 2 cytokine production and confers protection against obesity‐induced T2D [248]. Additionally, it has been reported that ILC2s in para‐aortic adipose tissue can mitigate atherosclerosis progression, and this athero‐protective role is potentially mediated by IL‐5 and IL‐13 [249]. However, a study by Sasaki et al. demonstrated a role for small intestinal ILC2s in promoting diet‐induced obesity, although the underlying mechanisms remain unclear [250]. In summary, the activation of specific signaling pathways in ILC2s represents a promising therapeutic avenue for ameliorating metabolic diseases. Nevertheless, the divergent effects observed across different tissues necessitate a deeper understanding of the contextual factors that determine ILC2 function to harness their full therapeutic potential.

Research has shown that ILC3s confer protection against HFD‐induced steatohepatitis via IL‐22 [251]. Upon activation by macrophage‐derived IL‐23, ILC3s secrete IL‐22, which in turn upregulates lipid metabolic pathways and suppresses hepatocyte apoptosis, ultimately alleviating NAFLD [251]. A recent study revealed that inhibiting VIPergic signaling in intestinal ILC3s enhances IL‐22 production and ameliorates hepatic steatosis [252]. This increased IL‐22 indirectly exerts its protective effect via the gut–liver axis, potentially by enhancing intestinal barrier function and modulating host lipid metabolism. Furthermore, IL‐22 exerts pleiotropic benefits on glucose regulation and lipid metabolism, demonstrating its therapeutic potential for treating metabolic dysfunctions such as diabetes, obesity, and steatohepatitis [129]. Consequently, targeting the ILC3–IL‐22 pathway represents a viable strategy to promote metabolic health.

In conclusion, research across diverse disease contexts has established ILCs as key integrators of environmental cues and central orchestrators of immunity. While the roles of ILCs extend beyond the conditions discussed here, the selection of cancer, chronic inflammation, and metabolic disorders in this review exemplifies the breadth of their functions—from antitumor surveillance and barrier defense to tissue repair and systemic metabolic regulation. The recurring themes of ILC plasticity, functional duality, and tissue‐specific programming affirm that their impact is not intrinsically beneficial or deleterious but is profoundly shaped by the contextual signals of the diseased tissue. The insights gleaned from these fields collectively enrich our understanding of ILC biology and provide a robust framework for re‐evaluating their roles in sepsis. Ultimately, viewing sepsis through the lens of ILC biology, as informed by these diverse diseases, may reveal novel pathways for immune intervention aimed at restoring balance in the dysregulated host response.

## 7 Therapeutic Strategies and Prospects for Targeting ILCs

Given the crucial roles of ILCs in the immune system and their involvement in various diseases, targeting ILCs is a promising novel biological approach. We summarize preclinical ILC‐based therapeutic strategies for sepsis that have been verified in vivo (Table 1) and discuss the clinical evidence for ILC‐targeting therapies in other inflammatory diseases (Table 2) to provide insights into sepsis. Supplementation with protective ILC effector cytokines is a direct and effective therapeutic strategy that has been verified in some animal experiments [135, 171, 173]. Furthermore, IFN‐γ and GM‐CSF, which are primary cytokines produced by ILC1s and ILC3s, have been demonstrated to alleviate immunosuppression in clinical trials [253, 254]. On the other hand, antibodies that target ILC‐derived cytokines or their receptors can help inhibit the pathogenic effects of ILCs [138, 180]. Emapalumab, which is a monoclonal antibody (mAb) against IFN‐γ, is currently undergoing clinical trials to evaluate the anticipated anti‐inflammatory benefits in patients with sepsis (NCT06694701). The efficacy of other antibodies, such as dupilumab (IL‐4Rα) [255], benralizumab (IL‐5Rα) [256], and seckukinumab (IL‐17A) [257], has been verified in clinical settings for a range of inflammatory diseases, providing new insights into the treatment of sepsis. Despite the therapeutic potential of cytokine‐based immunotherapy, significant challenges persist in practical applications, such as how to achieve specific delivery to target tissues or organs and achieve a satisfactory balance between pro‐ and anti‐inflammatory effects. Furthermore, systemic toxicity and the efficiency of cytokine metabolism require rigorous evaluation.

**TABLE 1:** Preclinical ILC‐targeted interventions for sepsis.

Therapeutic strategy	Subset	Drug/method	Sepsis model	Effect and mechanism	References
Supplementation with ILC effector cytokines	ILC2	IL‐9 (it)	CLP	Inhibits mTOR signaling; reduces macrophage apoptosis and M1 polarization; alleviates lung injury	[135]
	ILC2	IL‐4 (ip)	CLP	Activates STAT3 to upregulate LAMP2; enhances cardiac autophagosome–lysosome fusion and autophagy flux, ultimately increasing survival	[173]
	ILC2	IL‐13 (ip)	LPS (ip)	Induces STAT3 Ser727 phosphorylation; reduces mitochondrial‐mediated cardiomyocyte apoptosis, thereby improving cardiac function	[171]
Blockade of ILC effector cytokines	ILC1	Anti‐IFN‐γ antibody (ip)	LPS (ip)‐induced endotoxic shock	Elevates serum IL‐10; enhances septic shock tolerance; improves survival	[180]
	ILC2	Anti‐IL‐5 antibody (iv)	CLP	Reduces early pulmonary neutrophil and monocyte infiltration; lung injury not assessed	[138]
Promoting ILC activation	ILC2	NMU (ip)	CLP	Activates lung ILC2s to secrete IL‐9; promotes γδ T cell expansion and IL‐17A production; reduces mortality	[146]
	ILC2	IL‐33 pretreatment (it)	*Staphylococcus aureus* bacteremia	Activates lung ILC2s to secrete IL‐5/IL‐13; increases eosinophil recruitment; suppresses neutrophil infiltration; lowers bacterial load; improves survival	[43]
Suppressing ILC activation	ILC2	Soluble ST2 (ip)	CLP	Inhibits ILC2 activation and IL‐4/IL‐13 secretion; suppresses M2 macrophage polarization and Treg expansion; improves survival against secondary infections	[45]
	ILC2	Soluble ST2 (iv)	CLP	Decreases peritoneal IL‐10 and IL‐1β levels; reduces bacterial load and tissue damage (liver/ileum); transient survival benefit (72 h post CLP)	[44]
Modulating key transcription factors of ILC	ILC2	SR3335 (ip)	Severe burn injury	Inhibits RORα; suppresses intestinal ILC2 differentiation and maturation; reduces M2 macrophage polarization and bacterial translocation; improves survival	[133]
	ILC3	Astragalus/LYC‐55716 (ip)	CLP	Activates RORγt; promotes ILC3 proliferation and cytokine secretion; alleviates intestinal injury; reduces serum inflammatory cytokines; improves survival	[258]
	ILC2 and ILC3	IL‐7 (sc)	Two‐hit model (CLP followed by infectious pneumonia)	Enhances ILC proliferation and cytokine secretion; reverses immunosuppression; promotes pathogen clearance; increases survival	[259]
Modulating ILC migration	ILC2	AMD3100 (iv)	CLP	Inhibits CXCR4; promotes bone marrow‐to‐lung migration of ILC2; therapeutic efficacy not evaluated	[156]
Microbiome manipulation	ILC3	Fecal microbiota transplantation	*E*. *coli* K1 or *K. pneumoniae* (ip)‐induced sepsis	Activates intestinal ILC3s; induces IL‐17A‐mediated G‐CSF secretion; drives granulopoiesis and neutrophil recruitment; lowers bacterial load; improves survival	[57, 260]
	ILC3	Fecal microbiota transplantation	IRI‐induced enterogenic sepsis	Activates epithelial AHR; stimulates ILC3s to secrete IL‐22; enhances intestinal barrier integrity, reduces bacterial translocation, mitigates organ injury; improves survival.	[58]
Dietary intervention	ILC3	Enteral nutrition	CLP	Activates ILC3s; upregulates 5/15‐lipoxygenase expression; promotes resolvin D1–D5 production; alleviates intestinal inflammation; improves survival	[261]

Abbreviations: CLP, cecal ligation and puncture; IRI, ischemia–reperfusion injury; LPS, lipopolysaccharide; it, intratracheal injection; ip, intraperitoneal injection; iv, intravenous injection; sc, subcutaneous injection.

**TABLE 2:** Clinical ILC‐targeted interventions for inflammatory diseases.

Therapeutic strategy	Subset	Drug	Targeted disease	Therapeutic effect	Clinical trial	References
Blockade of ILC effector cytokines	ILC1	Emapalumab (anti‐IFN‐γ mAb)	Sepsis	Under evaluation	Phase IIa	NCT 06694701
	ILC2	Dupilumab (anti IL‐4/IL‐13 mAb)	Asthma	Improved lung function and reduced asthma exacerbations	Phase III	[255]
	ILC2	Benralizumab (anti IL‐5R mAb)	Asthma	Beneficial effects	Phase III	[256]
	ILC3	Secukinumab (anti IL‐17A mAb)	Active RA	Improved symptoms and reduced disease activity	Phase III	[257]
Suppressing ILC activation	ILC2	Tezepelumab (anti TSLP mAb)	Asthma	Lower exacerbation rates and improved lung function	Phase III	[256]
	ILC2	Itepekimab (anti IL‐33 mAb)	Asthma	Improved disease control and lung function	Phase II	[262]
	ILC3	Mirikizumab (anti IL‐23 mAb)	UC	Disease clearance and improved long‐term outcomes	Phase III	[263]
	ILC3	Ustekinumab (anti IL‐12/23 mAb)	CD	Beneficial effects	Phase III	[264]
Modulating ILC survival	ILC1	Tofacitinib (JAK inhibitor)	RA	Reduced pathogenic ILC1 frequency and IFN‐γ production	NA	[226]
	ILC2 and ILC3	CYT107 (rhIL‐7)	Sepsis	Reversed sepsis‐induced lymphopenia	Phase IIb	[265]
	ILC3	Tofacitinib (JAK inhibitor)	RA	Reduced PD1+ ILC3 and CTLA‐4+ ILC3 frequency; ameliorated the disease activity	NA	[227]

Abbreviations: mAb, monoclonal antibody; RA, rheumatoid arthritis; UC, ulcerative colitis; CD, Crohn's disease; NA, not applicable.

Immunotherapy targeting the upstream signals of ILCs can regulate their activation. For example, the administration of recombinant IL‐33 or NMU enhances the beneficial effect of ILC2s in septic mice [43, 146]. Conversely, neutralizing IL‐33 with soluble ST2, which is a decoy receptor that binds and inhibits IL‐33 signaling, limits ILC2‐mediated immunosuppression [44, 45]. Clinically, itepekimab (an IL‐33 mAb) has been shown to reduce the rate of exacerbation and improve lung function in patients with asthma [262]. However, given the ubiquitous expression of IL‐33 receptors across multiple immune cell types, strategies for selectively targeting ILC2s remain to be further explored. Furthermore, endogenous glucocorticoids increase mouse tolerance to septic shock by inhibiting the secretion of IFN‐γ from ILC1s, indicating a potential therapeutic approach. Glucocorticoids have been applied in patients with septic shock because of their potent anti‐inflammatory effects, although their clinical efficacy remains controversial [266]. Beyond glucocorticoid‐mediated suppression, pharmacological inhibition of the JAK/STAT pathway with tofacitinib represents a more direct strategy that has been shown to selectively target ILC1s, attenuating their frequency and proinflammatory function in patients with RA [226]. In addition, drugs that regulate the key TFs of ILCs can affect their development and function. SR3335, which is a specific inverse agonist of RORα, reverses the immunosuppressive role of ILC2s and mitigates gut bacteria‐associated sepsis by blocking the differentiation and maturation of ILC2s in mice [133]. Astragalus (an herbal medicine) and LYC‐55716 (a RORγt agonist) promote the proliferation and function of ILC3s by activating RORγt, thereby strengthening the intestinal immune barrier and improving inflammatory injury during sepsis [258]. IL‐7 is required for the development and maintenance of ILCs and regulates the activation of ILC‐related TFs [267]. Treatment with recombinant human (rhIL‐7) ameliorated sepsis‐induced depletion of ILC2s and ILC3s and significantly reduced mortality in septic model mice [259]. The administration of rhIL‐7 reversed lymphopenia and improved lymphocyte function in septic patients in a clinical trial [265]. PD‐1 is a checkpoint inhibitory molecule that is expressed on ILC2s. PD‐1 deficiency reverses sepsis‐induced impairment of IL‐13 production by ILC2s, mitigating sepsis‐associated muscle weakness in mice [178]. The PD‐1 inhibitor nivolumab has been investigated in Phase 1/2 trials in patients with sepsis‐induced immunosuppression, revealing preliminary evidence of its clinical efficacy and safety with no side effects, such as cytokine storm [268, 269]. In addition, modulating ILC migration directly influences their tissue‐specific immune functions. For example, AMD3100, which is a CXCR4 inhibitor, promotes the mobilization of ILC2ps from the BM to septic lungs in mice, thereby enhancing local immune defense [156].

Intestinal ILC activity is modulated by the gut microbiota and dietary cues, which may also constitute an effective strategy to enhance beneficial ILC‐mediated immune functions while suppressing detrimental hyperactive responses. Fecal microbiota transplantation has been confirmed to increase resistance to sepsis in mouse models by promoting ILC3 proliferation and cytokine secretion, although the specific microbial species responsible for this effect remain to be elucidated [57, 166, 260]. Dietary therapy, such as oral supplementation with *Lactobacillus acidophilus*, which is a common intestinal probiotic, can activate ILC3s and mitigate intestinal inflammation [270]. Recently, Hu et al. demonstrated that time‐restricted feeding alleviates liver injury in sepsis by regulating the intestinal flora, especially by reversing the reduction of Lactobacillus in septic mice [271]. In addition, Zhao et al. revealed that enteral nutrition alleviated intestinal inflammation and improved the survival rate in CLP mouse models by activating ILC3s, which upregulated 5‐ and 15‐lipoxygenase (enzymes for lipid mediator synthesis) to increase resolvin D1–D5 (inflammation‐resolving lipid molecules) production [261]. Thus, dietary interventions may synergize with microbial modulation to amplify ILC‐mediated protection.

In summary, therapeutic strategies targeting ILCs—ranging from cytokine modulation and receptor blockade to the regulation of upstream signals, key TFs, and even migration—present a promising yet complex frontier in the treatment of sepsis and other immune‐mediated diseases. However, the path to clinical translation is fraught with challenges, including achieving cell and tissue specificity, navigating the delicate balance between pro‐ and anti‐inflammatory effects, and ensuring long‐term safety. The lessons gleaned from targeting ILC‐related pathways in chronic inflammatory diseases provide valuable benchmarks, yet the hyperacute and dynamic nature of sepsis demands uniquely tailored solutions. Future success will likely depend on combinatory strategies that are informed by a deep understanding of the temporal dynamics of ILC responses during sepsis. Ultimately, moving beyond broad immunomodulation toward precise targeting of specific ILC subsets and their functional states holds the key to harnessing their full therapeutic potential, paving the way for a new class of immunotherapies that can restore immune homeostasis in critically ill patients.

## 8 Conclusion and Perspectives

ILCs have emerged as pivotal orchestrators of immunity, inflammation, and tissue homeostasis, extending far beyond their initial classification as innate counterparts of Th cells. This review has synthesized the current understanding of ILC biology, delineating their role in various diseases, with a particular focus on their complex, context‐dependent roles in sepsis. The functional duality of ILCs is a recurring theme: they are crucial for tissue homeostasis, pathogen clearance, and wound healing, yet their dysregulation can drive pathological inflammation, immune suppression, and tumor progression. The sepsis paradigm powerfully illustrates this duality, where, for instance, ILC2s can mediate both early lung protection through eosinophils recruitment and late‐phase immunosuppression, while ILC3‐derived IL‐22 can be either protective for the barrier or pathogenic when driving neutrophilic inflammation. This nuanced view challenges simplistic immunostimulatory or immunosuppressive therapeutic strategies and underscores the necessity for a more precise, ILC subset‐targeted approach.

Despite the increasingly recognized importance of ILCs, our understanding of the biology of these cells in sepsis remains nascent, with numerous unresolved questions. The precise mechanisms underlying the tissue‐specific roles of distinct ILC subsets and their dynamic contributions to organ injury or protection during sepsis warrant further elucidation. Notably, while ILC2 migration within the gut‒lung axis has been established, whether other ILC subsets exhibit significant migration during systemic inflammation remains an open question. The presence and functional scope of ILCregs, which is a recently identified cell subset, represent a notable gap in our knowledge and require validation in the context of sepsis and other diseases. The inherent plasticity and heterogeneity of ILCs present both a challenge for defining their exact roles and a promising opportunity for therapeutic modulation. A fundamental question is whether we are defining stable ILC lineages or transient functional states shaped by the tissue microenvironment; this distinction is critical for therapeutic targeting, as strategies for a stable lineage differ from those for a plastic state. Delineating how sepsis‐associated signals dictate ILC transdifferentiation holds significant potential for future research. Furthermore, as we highlight, ILCs may exert opposing effects on sepsis outcomes depending on the disease phase. This highlights the critical need for future studies to dissect the spatiotemporal specificity of ILC functions throughout the septic trajectory. Compounding these challenges, the majority of insights into ILCs are derived from mouse models. A significant translational gap exists because of the notable differences in the development, phenotype, and tissue distribution of ILCs between mice and humans. Studies in human are significantly limited by the tissue‐resident nature of ILCs, while analyses are largely confined to the peripheral blood of patients. Although alterations in circulating ILC subsets may correlate with disease progression, their exact clinical significance and potential utility as novel biomarkers demand rigorous validation. Consequently, the clinical relevance of targeting ILCs in human therapeutic settings remains to be firmly established.

The translation of ILC biology into effective therapies presents both immense promise and significant challenges. As reviewed, potential strategies span a wide spectrum, including cytokine supplementation, blockade of pathogenic cytokines, modulation of upstream activating signals, and exploitation of their inherent plasticity. However, the path to clinical application is fraught with obstacles. A primary challenge is the achievement of cell‐ and tissue‐specificity to avoid off‐target effects, given that receptors for key ILC‐modulating cytokines are widely expressed on various immune and stromal cells. This necessitates a deep understanding of the patient‐specific variables—including age, genetic background, comorbidities, microbiome composition, and the phase of the disease—that collectively shape an individual's immune response profile. The future lies in stratified medicine, where therapies are tailored not to a disease label, but to the precise immunological identity of the patient at a given moment.

To pave the way for such precision immunotherapies, future research must consider several key directions. First, there is an urgent need for longitudinal human studies that employ single‐cell and spatial omics technologies to map ILC dynamics across different diseases, from cancer and inflammatory conditions to metabolic disorders and acute critical illnesses such as sepsis. These studies must intentionally stratify patients based on the variables mentioned above to define clear ILC‐based endotypes. Second, the development of more sophisticated animal models that incorporate human‐relevant comorbidities and defined microbial consortia is crucial for mechanistic discovery and therapeutic testing. Third, exploring the crosstalk between ILCs and the nervous system, the microbiota, and the metabolic machinery will reveal novel regulatory axes that can be therapeutically harnessed. Finally, the potential of ILCs as dynamic biomarkers for disease prognosis and treatment response should be rigorously evaluated to bridge the gap between bench‐side discovery and bedside application.

In conclusion, ILCs are central integrators of environmental cues and orchestrators of immune responses across health and disease. The insights gleaned from studying ILCs in sepsis provide a powerful lens through which to view their core characteristics—swift responsiveness, functional plasticity, and extensive cellular crosstalk—principles that are universally applicable to understanding their roles in other pathological states. Consequently, the pivotal challenge and opportunity in the field lie in advancing from nonspecific immune modulation to the precise, context‐informed targeting of specific ILC subsets and their functional states. The ultimate goal is to harness the full therapeutic potential of these versatile cells to restore immune homeostasis, not only in sepsis but also in a broad spectrum of human diseases, thereby translating the complexities of ILC biology into tangible benefits for patients.

## Author Contributions

**Zhenzhen Zhan**: writing – original draft. **Heyang Sun, Chenning Li, and Qianya Hong**: writing – review and editing and visualization. **Shuainan Zhu and Ying Yu**: resources and investigation. **Hao Zhang and Kefang Guo**: conceptualization, supervision, and funding acquisition. All authors have read and approved the final manuscript.

## Ethics Statement

The authors have nothing to report.

## Conflicts of Interest

The authors declare no conflicts of interest.

## Data Availability

The authors have nothing to report.
